# Release of Volatile Cyclopentanone Derivatives from Imidazolidin-4-One Profragrances in a Fabric Softener Application

**DOI:** 10.3390/molecules28010382

**Published:** 2023-01-02

**Authors:** Serge Lamboley, Basile Vuichoud, Jean-Yves de Saint Laumer, Andreas Herrmann

**Affiliations:** Division Recherche et Développement, Firmenich SA, Rue de la Bergère 7, 1242 Satigny, Switzerland

**Keywords:** cleavage reactions, delivery systems, fragrances, heterocycles, hydrolysis, perfumery, properfumes, stereochemistry

## Abstract

Imidazolidin-4-ones were investigated as hydrolytically cleavable profragrances to increase the long-lastingness of perfume perception in a fabric softener application. The reaction of different amino acid amides with 2-alkyl- or 2-alkenylcyclopentanones as the model fragrances to be released afforded the corresponding bi- or tricyclic imidazolidin-4-ones as mixtures of diastereoisomers, which were separated by column chromatography. In polar solution, the different stereoisomers equilibrated under thermodynamic conditions to form mixtures with constant isomeric distributions, as shown by NMR spectroscopy. Dynamic headspace analysis on dry cotton demonstrated the controlled fragrance release from the precursors in practical application. Under non-equilibrium conditions (continuous evaporation of the fragrance) and depending on the structure and stereochemistry of the profragrances, the recorded headspace concentrations of the fragrance released from the precursors increased by a factor of 2 up to 100 with respect to the unmodified reference. Prolinamide-based precursors released the highest amount of fragrance and were thus found to be particularly suitable for prolonging the evaporation of cyclopentanone-derived fragrances on a dry cotton surface.

## 1. Introduction

Fragrances are key ingredients for perfumed consumer articles, such as body care and household products [1]. The pleasantness of the perfume influences the choice for a given product and, at the same time, the performance of perfumed consumer articles is often associated with the longevity of fragrance perception in application [2]. However, fragrances are highly volatile and thus rapidly evaporate from the surfaces they have been applied to, which considerably limits the duration of the smell. Achieving a long-lasting perfume impression in functional perfumery applications is therefore an important research target in the fragrance industry. 

The long-lastingness of fragrances can be increased either through encapsulation in various polymer systems and release by mechanical rupture of the capsules [3,4,5,6,7,8,9,10,11] or, alternatively, generation from so-called profragrances or properfumes by covalent bond cleavage from a suitably designed precursor [11,12,13,14,15,16]. To perform under everyday conditions, covalent bonds have to be cleaved under mild conditions that are encountered in the environment, such as hydrolysis (e.g., induced by a change of pH), slow oxidation in ambient air, small temperature changes, or the presence of enzymes or light. Hydrolytically cleavable profragrances represent a particular challenge because they are expected to be stable during storage in an aqueous environment, but to efficiently release the fragrance during application [12]. 

Imidazolidin-4-ones have attracted some attention in medicinal chemistry [17], e.g., for their antiviral properties [18]. Furthermore, like other 1,3-heterocycles, they are hydrolysed in aqueous media to form an amino acid amide together with a carbonyl compound, which led to their investigation as prodrugs to deliver peptides [19,20,21,22] and various amino acid or peptide derivatives with pharmaceutical properties [23,24,25,26,27,28,29,30]. The structure of the carbonyl compound has been adjusted to optimise the kinetics for the efficient release of the amino acid amide. 

**Scheme 1:**
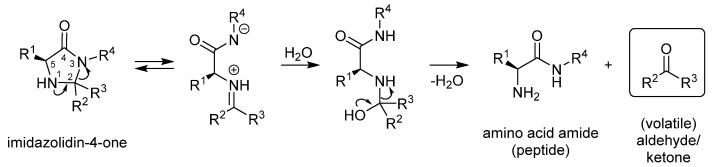
Mechanism of the hydrolysis of imidazolidin-4-ones to form amino acid amide or peptide derivatives and carbonyl compounds at neutral pH [23].

Under physiological conditions (phosphate buffer, pH 7.4), which are relevant for pharmaceutical and some perfumery applications, the hydrolysis of imidazolidin-4-ones has been reported to follow an S_N_1-type mechanism with C(2)–N(3) bond cleavage to generate the targeted amino acid amide or peptide derivative, together with a carbonyl compound, as outlined in Scheme 1 [23]. Studies over a broader pH range revealed first-order kinetics in acidic solution. Typically, pH-rate profiles with sigmoidal shapes have been recorded with maximum rates above pH 4 [22,23,24,25]. Furthermore, kinetic measurements showed the rates to be independent of the buffer concentration, which indicated the absence of general acid or base catalysis [20,21,22,23,24,25]. Both protonated (at acidic pH) and neutral species (under physiological conditions) undergo spontaneous hydrolysis, with the reaction at acidic pH being slower than at neutral pH [23,24]. The structures of the amino acid substituent R^1^, the carbonyl group functions R^2^ and R^3^ and the amide group R^4^ influence the rate of hydrolysis (Scheme 1). 

It is not evident that knowledge about the development of prodrugs can be transferred to profragrances, as different requirements for the release of the active compound must be met in the two cases. First, in contrast to prodrugs, profragrances should generate volatile compounds as the active fragrances to be released. In the example of imidazolidin-4-one-derived profragrances, this would be carbonyl compounds, and the non-volatile amino acid amine structure could be varied to adjust the rate of hydrolysis. Second, the imidazolidin-4-ones have to efficiently be deposited onto a target surface during application, from which the released fragrance should evaporate. Finally, all perfumed body care or household articles contain surfactants, the presence of which influences both the deposition of the profragrance onto the surface and the rate of fragrance release. 

**Scheme 2:**
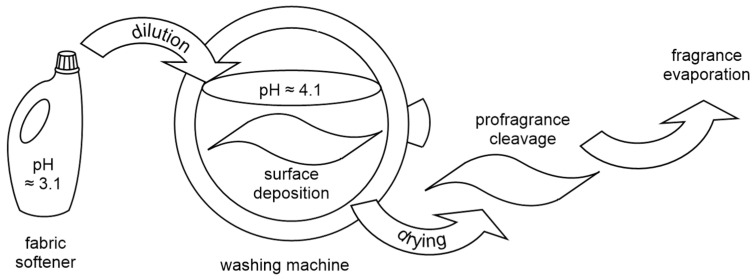
Simplified fabric softening process in a washing machine. Profragrances are indirectly deposited onto fabric by partitioning between the diluted softener emulsion and the fabric surface. Profragrances are expected to release the volatile fragrance after dilution, surface deposition and drying.

Rinse-added fabric softeners represent a typical application in which profragrances are commonly used to increase the long-lastingness of fragrance perception. A generalised fabric softening process is illustrated in Scheme 2. The perfume and the profragrance are formulated in a concentrated fabric softener emulsion containing a cationic surfactant that is responsible for the softening effect. Typical cationic surfactants used in fabric softeners are quaternised triethanolamine esters of fatty acids (triethanolamine esterquats) [31,32]. The concentrated formulation has a pH of ca. 3.1, at which the profragrance should have maximum stability. After the washing cycle, the softener formulation is diluted, which increases the pH by about one unit to reach a value of ca. 4.1. The perfume and the properfume are then deposited onto the fabric by partitioning between the aqueous surfactant emulsion and the textile surface (indirect deposition) [33,34]. After a few minutes, the non-deposited material is removed with the surfactant emulsion, and the fabric is dried. Profragrance cleavage should occur on the dry fabric to ensure a long-lasting fragrance release. Successful profragrances are thus expected to be stable during storage in the concentrated product formulation, efficiently deposited onto the fabric surface in the softening process and cleaved under environmental conditions on the dry surface to generate the fragrance. 

We have previously investigated the controlled release of fragrance aldehydes and ketones from imidazolidin-4-ones in fabric softener applications [35]. In that study, we demonstrated that under realistic application conditions, the release of fragrance ketones was considerably more efficient than that of aldehydes, but that neither the relative release kinetics in solution nor the hydrophobicity of the imidazolidin-4-one profragrance (which influences deposition) allowed prediction of the measured release efficiency in application [35]. 

**Figure 1:**
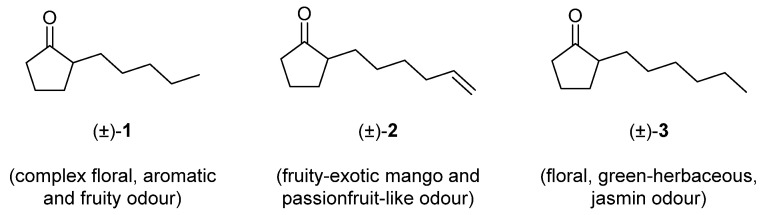
Structures and olfactive descriptors of cyclopentanone-derived fragrances (±)-**1**–(±)-**3**.

Volatile 2-alkyl- and 2-alkenylcyclopentanones such as (±)-**1**–(±)-**3** (Figure 1) with fruity and/or floral olfactive characteristics [36,37,38] represent an interesting family of perfume materials to be released from profragrances [39]. The goal of the present work was to evaluate whether hydrolytically cleavable imidazolidine-4-one profragrances could prolong the longevity of cyclopentanone fragrances in functional perfumery applications. We thus prepared a series of imidazolidin-4-one-based profragrances of (±)-**1**–(±)-**3** derived from different amino acid amides, separated the different diastereoisomers of the bi- or tricyclic imidazolidine-4-one profragrances and analysed their reversible isomerisation under thermodynamic conditions. We then investigated how the structure of the profragrances influenced the efficiency of fragrance release on dry cotton in a fabric softener application. 

## 2. Results and Discussion

### 2.1. Synthesis and Characterisation of Imidazolidin-4-One-Based Profragrances

**Scheme 3:**
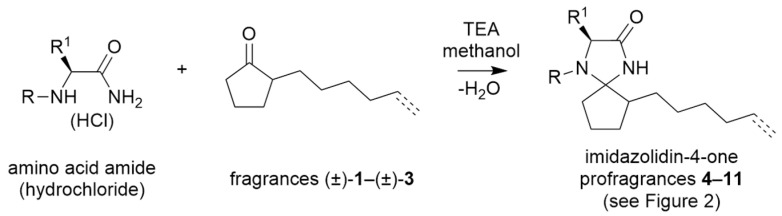
One step synthesis of imidazolidine-4-one profragrances **4**–**11** from amino acid amides.

**Figure 2:**
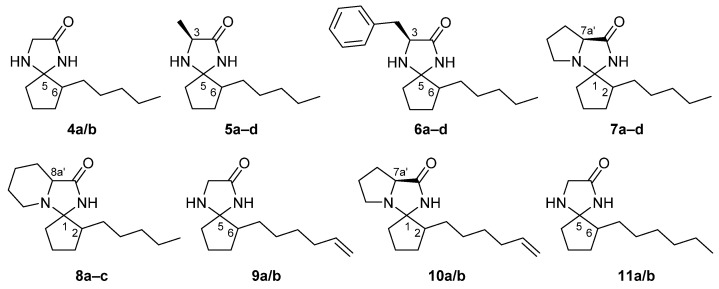
Structures of profragrances **4**–**11** releasing cyclopentanones (±)-**1**–(±)-**3** by hydrolysis. The formation of the heterocycle generates a new stereocentre at the C(1) or C(5) positions, thus all compounds were obtained as mixtures of diastereoisomers (designated by the letters **a**–**d**). All stereocentres are labelled with the corresponding atom numbers.

Imidazolidin-4-one derivatives are readily prepared in one step by reaction of amino acid amides or their corresponding hydrochlorides with carbonyl compounds in refluxing methanol. The reaction is carried out in the presence of triethylamine (TEA) and, optionally, molecular sieves (Scheme 3) [20,23,24,29,35]. We thus prepared compounds **4**–**11** (Figure 2) by condensation of racemic 2-alkyl- or 2-alkenylcyclopentanones (±)-**1**–(±)-**3** with the commercially available hydrochloride salts of glycinamide, alaninamide, phenylalaninamide and prolinamide or with 2-piperidinecarboxamide [39]. 

The formation of the heterocycle in the reaction of amino acid amides with cyclopentanone derivatives generated a new stereocentre at C(5) of 1,4-diazaspiro[4.4]nonan-2-ones **4**–**6**, **9** and **11** (bicyclic structures), and at C(1) of tetrahydrospiro[cyclopentane-1,3′-pyrrolo[1,2-*c*]imidazol]-1′(2′*H*)-ones **7** and **10** or tetrahydro-2′*H*-spiro[cyclopentane-1,3′-imidazo[1,5-*a*]pyridin]-1′(5′*H*)-one **8** (tricyclic structures). Profragrances **4**–**11** were thus obtained as mixtures of diastereoisomers. With glycinamide being achiral, a racemic pair of diastereoisomers was obtained in the case of profragrances **4**, **9** and **11**. Use of amino acid amides other than glycinamide, e.g., for the preparation of **5**–**8** and **10**, generated an additional stereocentre to the molecule, giving rise to a set of four different diastereoisomers. If the amino acid amides are derived from the natural *l*-amino acids, this additional stereocentre is always in the (*S*)-configuration (except for *l*-cysteine).

**Scheme 4:**
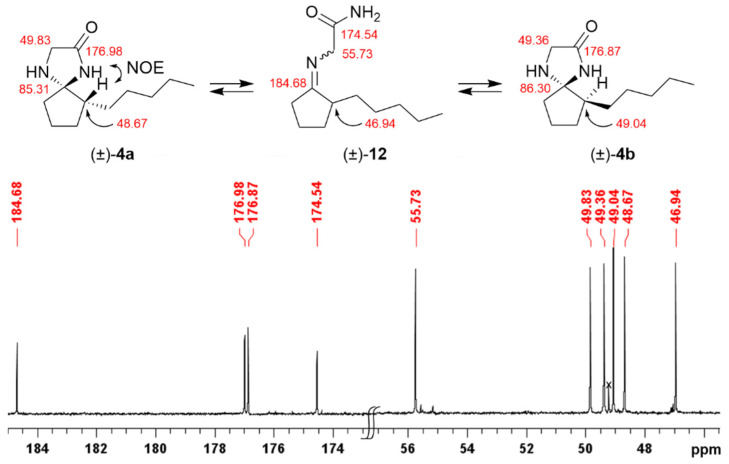
Expansion of the ^13^C NMR spectrum (CDCl_3_) of the crude product obtained from the reaction of glycinamide hydrochloride with (±)-**1** and structural assignment of (5*RS*,6*RS*)-**4a**, (5*RS*,6*SR*)-**4b** and imine (±)-**12**, obtained in an approximative ratio of 1:1:1 (the peak labelled with x corresponds to remaining (±)-**1**).

Reaction of equimolar amounts of glycinamide hydrochloride and (±)-2-pentylcyclopentan-1-one ((±)-**1**) yielded, after column chromatography, about 30–35% of profragrance **4a**/**b** as a 1:1 mixture of two racemic diastereoisomers. Using an excess of glycinamide hydrochloride (2 eq.) increased the isolated yield to 48%. Analysis of the crude reaction product (before chromatography) showed four singlets (184.7, 177.0, 176.9 and 174.5 ppm) in the region between 185 and 174 ppm of the ^13^C NMR spectrum (Scheme 4), two of them corresponding to C(2) of the two diastereoisomers of 1,4-diazaspiro[4.4]nonan-2-ones (±)-**4a** and (±)-**4b** (177.0 and 176.9 ppm). Furthermore, the spectrum displayed three triplets (55.7, 49.8 and 49.4 ppm) and three doublets (49.0, 48.7 and 46.9) in the region between 56 and 46 ppm. The different peaks were all of similar intensity, indicating the presence of three products in an approximative ratio of 1:1:1. Because the spectrum displayed only two singlets at 86.3 and 85.3 ppm, which are characteristic for C(5) of the 1,4-diazaspiro[4.4]nonan-2-ones, the third product was assigned to imine (±)-**12**, with the signal at 184.7 ppm corresponding to the imine group.

Imine (±)-**12** is expected to be formed as an intermediate in the reaction [26], but its presence after several hours of reflux in methanol suggested that its cyclisation to the imidazolidine-4-one was slow. Column chromatography efficiently removed the imine from the product, presumably due to its hydrolysis to glycinamide and (±)-**1** on the stationary phase, to afford a mixture of (±)-**4a**/**b**. With (±)-**12** representing one third of the crude product, this might explain why 1,4-diazaspiro[4.4]nonan-2-ones (±)-**4a**/**b** were isolated in yields only below 50%.

When we eluted (±)-**4a**/**b** at lower polarity, column chromatography allowed the separation of the two diastereoisomers (±)-**4a** and (±)-**4b**, which were obtained in purities above 90%. In the case of (±)-**4a**, NOESY analysis showed a correlation between the protons at N(1) and C(6); therefore its structure was assigned as (5*RS*,6*RS*)-**4a**. Consequently, the other isomer was designated as (5*RS*,6*SR*)-**4b** (Scheme 4).

**Figure 3:**
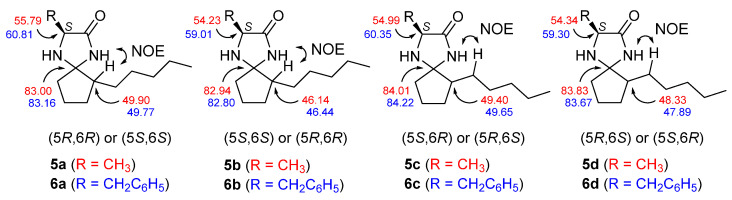
Characteristic ^13^C NMR chemical shifts and structural assignment of the diastereoisomers of *l*-alaninamide-derived profragrances **5a**–**d** (R = CH_3_, red) and of *l*-phenylalanine-derived profragrances **6a**–**d** (R = CH_2_C_6_H_5_, blue).

Reacting (±)-**1** with two equivalents of *l*-alaninamide hydrochloride under the same conditions afforded profragrances **5a**–**d**. Integrating the signals of C(3) situated between 54 and 56 ppm in the ^13^C NMR spectrum of crude **5** (before column chromatography) showed the formation of four diastereoisomers **5a** (55.8 ppm), **5b** (54.2 ppm), **5c** (55.0 ppm) and **5d** (54.3 ppm) in a ratio of 24%:20%:26%:26% (Figure 3), together with only 4% of the corresponding non-cyclised imine (two signals around 61.3 ppm). Column chromatography afforded a mixture of **5a**–**d** in 84% isolated yield. The higher yield with respect to the previous reaction with glycinamide suggested that the introduction of the additional methyl group reduced the amount of imine found in the crude product and thus increased the overall yield of the target compounds.

The different diastereoisomers could be only partially separated. Repetitive column chromatography allowed the isolation of **5a** and **5d** in purities >95%; **5b** was characterised as the minor isomer in a mixture with **5a**, and **5c** as the major isomer in a mixture with **5d**.

Observation of an NOE between the protons at N(1) and C(6) indicated the same relative stereochemistry (3*S*,5*R*,6*R* or 3*S*,5*S*,6*S*) for structures **5a** and **5b**. Similarly, a correlation between the protons at N(1) and C(1) of the pentyl chain was observed in the NOESY spectra of **5c** and **5d**, confirming that these two structures also had the same relative stereochemistry (3*S*,5*S*,6*R* or 3*S*,5*R*,6*S*). The absolute stereochemistry of the four diastereoisomers could not unambiguously be determined by NMR spectroscopy.

We then also prepared profragrances **6a**–**d** by reaction of *l*-phenylalaninamide hydrochloride with (±)-**1**. Analysis of the crude reaction product showed that the sample was composed of **6a**–**d** in a ratio of 21%:20%:22%:27% and containing about 10% of the intermediate imine. Column chromatography afforded two product fractions, each containing two pairs of diastereoisomers, namely **6a** and **6c** (ca. 50%:50%) and **6b** and **6d** (ca. 45%:55%), which were not further separated. ^13^C NMR analysis showed similar chemical displacements for the signals at C(5) and C(6) of phenylalaninamides **6a**–**d** as the alaninamide analogues **5a**–**d** discussed earlier. The structures of **6a**–**d** (with relative stereochemistry) were thus assigned in analogy, as shown in Figure 3.

**Figure 4:**
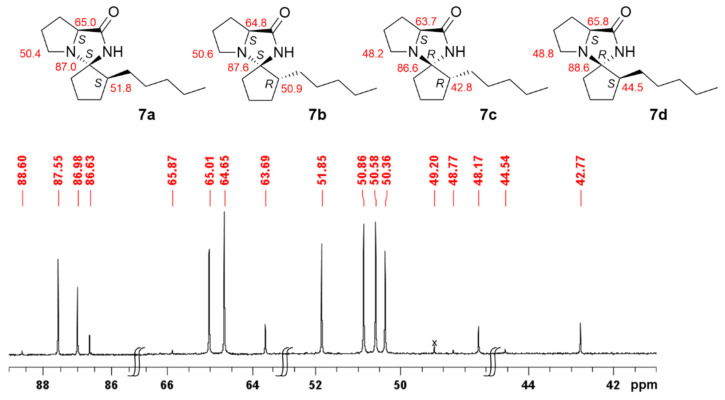
Expansion of the ^13^C NMR spectrum (CDCl_3_) of the crude product obtained from the reaction of *l*-prolinamide hydrochloride with (±)-**1** and structures of (1*S*,2*S*,7a′*S*)-**7a**, (1*S*,2*R*,7a′*S*)-**7b**, (1*R*,2*R*,7a′*S*)-**7c** and (1*R*,2*S*,7a′*S*)-**7d** obtained in an approximative ratio of 18:26:5:1 (the peak labelled with x corresponds to remaining (±)-**1**). The structural assignment of the diastereoisomers was based on calculated relative energy differences.

Reaction of *l*-prolinamide hydrochloride with (±)-**1** afforded the crude reaction product in 81% yield. Four possible diastereoisomers of tricyclic tetrahydrospiro[cyclopentane-1,3′-pyrrolo[1,2-*c*]imidazol]-1′(2′*H*)-ones **7a**–**d** bearing three five-membered rings can be formed as depicted in Figure 4. Because of the cyclic proline structure, the formation of the intermediate imine structure (equivalent to (±)-**12**), is not possible. This might explain the higher product yield with respect to the reactions of the other amino acid amides described earlier. Furthermore, the ^13^C NMR spectrum of the crude product mixture (Figure 4) showed that the different diastereoisomers were not obtained in equimolar amounts, but in a ratio of **7a**/**7b**/**7c**/**7d** ≈ 36%:52%:10%:2%, which is presumably due to the formation of a strained tricyclic structure giving rise to energetically more or less favourable isomers.

The structures of the different diastereoisomers of **7a**–**d**, as shown in Figure 4, were determined by calculating the relative energies of the different diastereoisomers by using the density functional theory (DFT) [40,41] (Table 1). Conformers of each stereoisomer were determined by using the mechanic force field MMFF94s as implemented in MacroModel [42], and the 16 lowest energy conformers were then re-optimised at the DFT level with the ωB97X-D/6-31G* method in the gas phase [43] to determine the minimum energy. On the basis of these calculations, diastereoisomers (1*S*,2*S*,7a′*S*)-**7a** and (1*S*,2*R*,7a′*S*)-**7b** represented the major compounds in the mixture if the reaction is controlled by a thermodynamic equilibrium.

**Figure 5:**
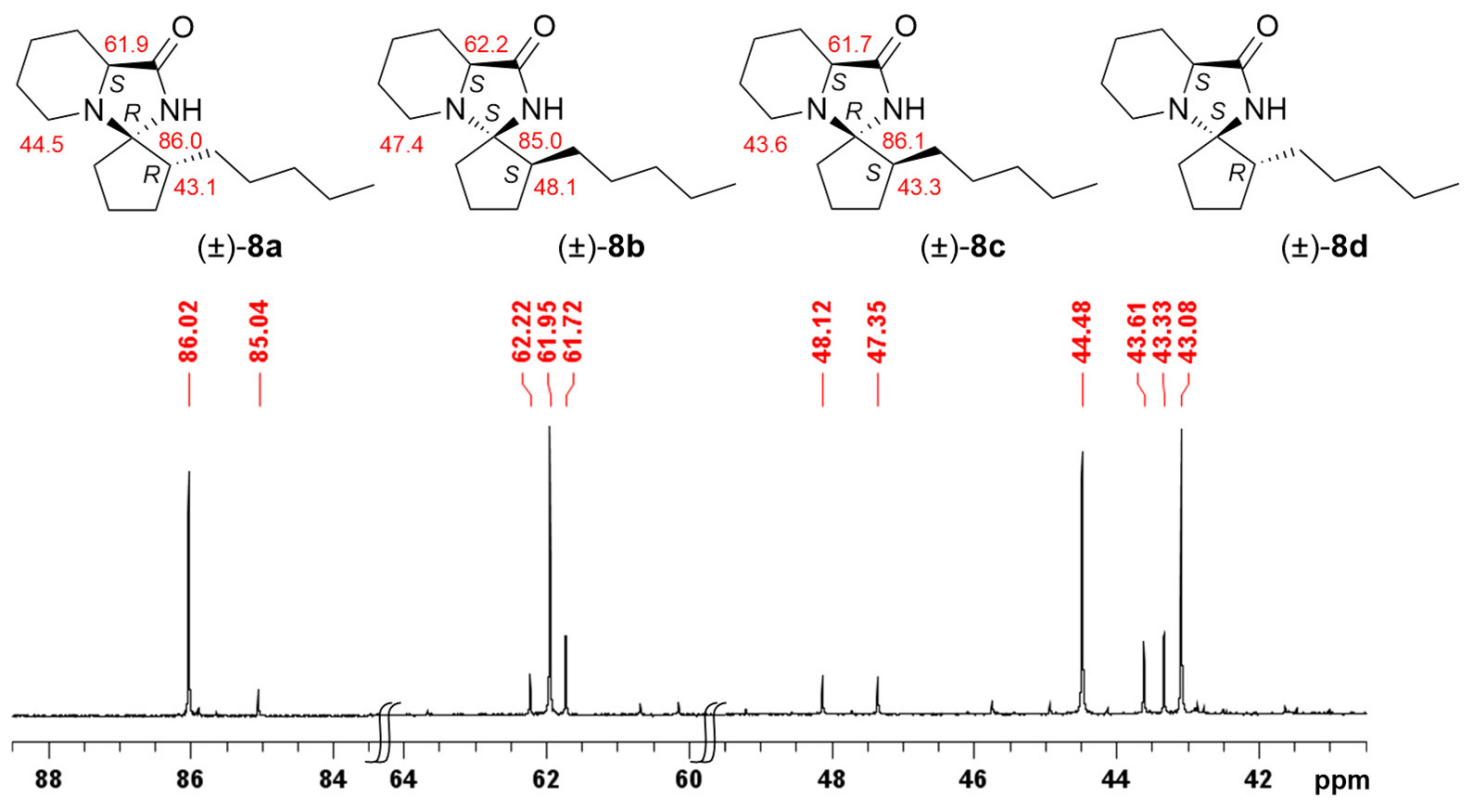
Expansion of the ^13^C NMR spectrum (CDCl_3_) of the crude product obtained from the reaction of (±)-2-piperidinecarboxamide with (±)-**1** and structures of (1*RS*,2*RS*,8a′*SR*)-**8a**, (1*SR*,2*SR*,8a′*SR*)-**8b** and (1*RS*,2*SR*,8a′*SR*)-**8c** obtained in an approximative ratio of 71:10:19 (only one of the enantiomers is depicted). The structural assignment of the diastereoisomers was based on calculated relative energy differences.

Reaction of racemic 2-piperidinecarboxamide with (±)-**1** formed tricyclic profragrances (±)-**8a**–**d** bearing one six-membered and two five-membered rings (Figure 5). The reaction can yield four possible diastereoisomers, all of which are obtained as racemates. The reaction of (±)-**1** with 2-piperidinecarboxamide seemed to be less efficient than with *l*-prolinamide, the crude reaction product was obtained in only 40% yield. As observed for the prolinamide derivatives described earlier, the different diastereoisomers of **8** were not formed in equimolar quantities, but in a ratio of **8a**/**8b**/**8c** ≈ 71%:10%:19% (Figure 5). The presence of the fourth diastereoisomer, **8d**, could not unambiguously be determined in the reaction mixture. A series of small signals in the ^13^C NMR spectrum of the crude compound, e.g., in the region between 48 and 42 ppm, might indicate its formation at less than 5%. Column chromatography afforded (±)-**8a**, a mixed fraction of (±)-**8a** and (±)-**8b** (ca. 44%:56%) and (±)-**8c**.

The structural assignment of (±)-**8a**–**d** was based on DFT calculations, as outlined earlier (Table 1). Diastereoisomer **8a** was isolated as the major compound of the reaction and was thus assigned to the (1*RS*,2*RS*,8a′*SR*)-isomer, which corresponded to the lowest calculated relative energy. Inversely, (1*SR*,2*RS*,8a′*SR*)-**8d** was calculated to have the highest relative energy (+22.9 kJ mol^–1^), which explained why this isomer was formed only in trace amounts or not at all. The relative energies of the other two diastereoisomers (1*SR*,2*SR*,8a′*SR*)-**8b** and (1*RS*,2*SR*,8a′*SR*)-**8c** were relatively close, and their structures were tentatively assigned according to the calculated energy differences (Table 1).

Finally, we prepared imidazolidine-4-one-derived profragrances **9**–**11** of 2-(5-hexenyl)cyclopentan-1-one ((±)-**2**) and 2-hexylcyclopentan-1-one ((±)-**3**) by reaction with glycinamide or *l*-prolinamide hydrochloride. Again, column chromatography allowed partial separation of the different diastereoisomers, the structures of which were assigned by NMR spectroscopy in analogy to 1,4-diazaspiro[4.4]nonan-2-ones **4a**/**b** (for **9a**/**b** and **11a**/**b**) and tetrahydrospiro[cyclopentane-1,3′-pyrrolo[1,2-*c*]imidazol]-1′(2′*H*)-ones **7a**/**b** (in the case of **10a**/**b**). In the case of **11**, the two diastereoisomers **11a** and **11b** were not separated.

### 2.2. Isomerisation of Profragrances ***4a***/***b*** and ***7a***–***c*** in Methanol

**Scheme 5:**
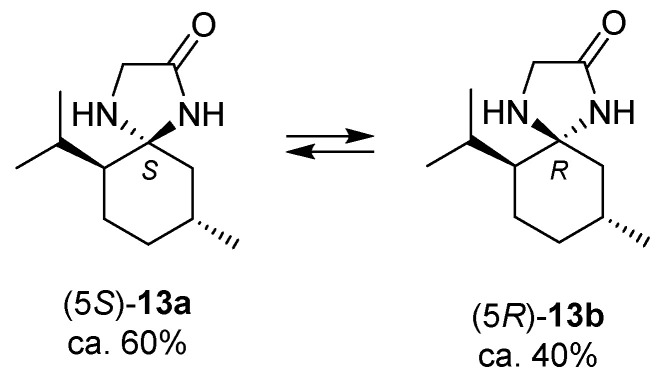
Equilibration of glycinamide-derived profragrances **13a**/**b** in methanol [35].

We have previously demonstrated that menthone-releasing imidazolidine-4-ones **13a**/**b** (Scheme 5) isomerised in protic solvents or in the presence of a proton source to reach a thermodynamic equilibrium between the two diastereoisomers [35]. NMR measurements of either (5*S*)-**13a** or (5*R*)-**13b** in methanol for several days showed that a mixture of the two compounds in a ratio of ca. 3:2 was reached.

**Scheme 6:**
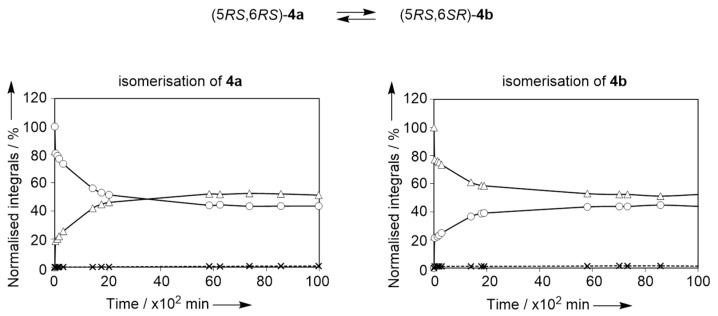
Isomerisation of profragrances **4a** (––**○**––) and **4b** (––Δ––) via intermediate (±)-**12** (not shown) and slow formation of glycinamide (---×---) in methanol-d_4_ as observed by ^1^H NMR spectroscopy for ca. 10,000 min.

**Scheme 7:**
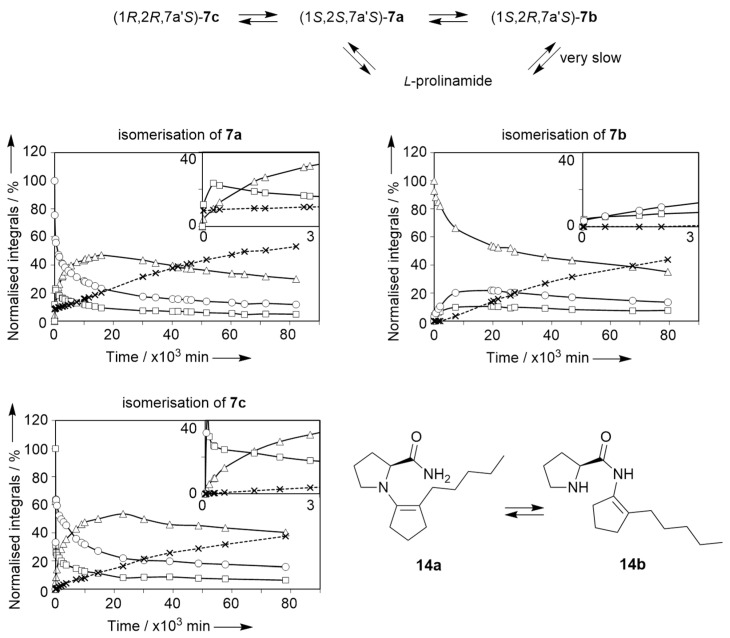
Isomerisation of profragrances **7a** (––**○**––), **7b** (––Δ––) and **7c** (––**□**––) via intermediates **14a**/**b** and slow formation of *l*-prolinamide (---×---) in methanol-d_4_ as observed by ^1^H NMR spectroscopy for ca. 80,000 min. The inserts show the evolution of the isomerisation during the first 3000 min.

Having isolated the pure diastereoisomers of glycinamide-derived profragrances (5*RS*,6*RS*)-**4a** and (5*RS*,6*SR*)-**4b**, and of *l*-prolinamide-derived profragrances (1*S*,2*S*,7a′*S*)-**7a**, (1*S*,2*R*,7a′*S*)-**7b** and (1*R*,2*R*,7a′*S*)-**7c**, we wondered whether they also isomerised in a polar environment. We thus performed ^1^H NMR measurements of the individual diastereoisomers in methanol-d_4_ over a period of about 50,000 min (ca. 35 d, **4**) and 80,000 min (ca. 55 d, **7**). Scheme 6 and Scheme 7 show the amount of the different isomers present in the mixture at different time points. Numerical data for Scheme 6 and Scheme 7 and for the following figures are provided in the Supplementary Material. For the measurements, the protons at C(3) of **4a** (3.44, 3.40, 3.32, 3.29 ppm) and **4b** (3.42, 3.38, 3.37 and 3.34 ppm, although the quartets overlapped, they could be properly integrated), as well as at C(7a′) of **7a** (3.71–3.79 ppm), **7b** (3.85–3.90 ppm) and **7c** (3.80–3.85 ppm), were integrated, together with the corresponding protons of glycinamide (3.81–3.83 ppm), imine (±)-**12** (3.84–3.86 ppm) and *l*-prolinamide (3.65–3.71 ppm). The sum of these integrals was considered as 100% at each time point.

As previously observed for (5*S*)-**13a** and (5*R*)-**13b**, the pair of diastereoisomers (±)-**4a** and (±)-**4b** obtained from the reaction of glycinamide with (±)-**1** isomerised to form an equilibrium of the two compounds in a ratio of ca. 45:55 after ca. 8000 min (about 5.5 d) (Scheme 6). Only small (but slowly increasing) amounts of glycinamide (ca. 1–2%) and imine (±)-**12** (ca. 2–4%) were detected in the ^1^H NMR spectra. Both isomers thus hydrolysed very slowly in methanol.

In the case of the *l*-prolinamide derivative of (±)-**1**, three of the four expected diastereoisomers (**7a**–**c**) were isolated in sufficient quantities for the NMR studies. The data in Scheme 7 show that they isomerised to reach a ratio of approximately 25:64:11 at the end of the experiment (after ca. 80,000 min), with the thermodynamically most stable isomer (1*S*,2*R*,7a′*S*)-**7b** being the major component, followed by (1*S*,2*S*,7a′*S*)-**7a** and the least stable isomer (1*R*,2*R*,7a′*S*)-**7c**. Isomer **7c** was rapidly transformed to **7a**, which was then slowly converted to **7b**. The isomerisation of the diastereoisomers was found to be reversible in all cases (**7a** can form **7c**, and **7b** can form **7a**, see inserts in Scheme 7). To explain the isomerisation at C(2) from *R* (in **7c**) to *S* (in **7a** and **7b**) we assumed that the isomerisation passed through intermediates **14a** and/or **14b** (Scheme 7) with a double bond between C(1) and C(2). No evidence for the presence of intermediate **14a**/**b** was found in the NMR analyses, which indicated that the transition through these structures must be rapid.

From our NMR data, we could also see that the hydrolysis of the profragrances to form *l*-prolinamide occurred fast from **7a** at high concentration (more than 8.5% of *l*-prolinamide were already formed after only 32 min) and that hydrolysis of the most stable isomer **7b** was the slowest of the observed reaction steps. With the hydrolysis of **4a**/**b** to form glycinamide being very slow, the rapid formation of *l*-prolinamide form **7a**–**c** might be due to the release of strain from the tricyclic structures. Under thermodynamic conditions (i.e., if no compound is removed from the equilibrium), the hydrolysis of the imidazolidin-4-one must be reversible to eventually reach a constant amount of *l*-prolinamide in the mixture.

Although the isomerisations of **4a**/**b** and **7a**–**c** proceeded differently, constant equilibria corresponding to the thermodynamic mixture should finally be reached in all our measurements. The reversible formation and hydrolysis of imidazolidine-4-ones resembles that of previously described imidazolidines (aminals), which were obtained from a mixture of aldehydes and a diamine [44,45]. These previous studies have shown that the formation of thermodynamic equilibria from so-called dynamic mixtures [46] can be efficient for the controlled slow release of fragrances in application. In closed systems, when the fragrances cannot evaporate, a stable equilibrium is reached. Upon application, the equilibrium is shifted by the slow evaporation of the fragrance, achieving a long-lasting fragrance perception. In the next section, we describe how we investigated the release of cyclopentanones (±)-**1**–(±)-**3** from their respective profragrances on cotton under application conditions.

### 2.3. Dynamic Headspace Analysis on Dry Cotton after a Typical Fabric Softening Application

The performance of the different profragrances was evaluated by monitoring the fragrance release by dynamic headspace analysis on dry cotton after a typical fabric softening process (Scheme 2) [35,47,48]. Previous work on prodrugs indicated that, because of the decrease of internal strain during hydrolysis, imidazolidin-4-ones derived from cyclopentanones hydrolysed more rapidly than other linear or cyclic ketones [20,21,23,24]. On the other hand, it has been reported that protonated imidazolidin-4-ones derived from cyclopentanones are about 200 times less reactive than the corresponding neutral form [23]. We therefore hoped that cyclopentanone-releasing imidazolidine-4-one profragrances might be suitable for fabric softener applications, with the highly acidic pH of the concentrated formulation slowing the hydrolysis of the precursors during storage and an increase of pH during application accelerating the release of the fragrance ketones.

In a simplified laboratory protocol [49], profragrances **4**–**11** were dispersed in a concentrated triethanolamine esterquat fabric softener formulation (at 0.014 mmol g^−1^ of softener). An aliquot of the fabric softener was then weighed into a small bottle and diluted with water by a factor of ca. 330. A cotton sheet (square of 15 × 15 cm) was added, and the sample was shaken for 3 min, left standing for 2 min and then wrung out and line dried. Reference samples containing an equimolar amount of the fragrance to be released were prepared accordingly.

For the headspace measurements, the line-dried cotton sheets were placed inside a home-made sampling cell with an inner volume of ca. 165 mL. The sampling cells were thermostatted (25 °C) and a constant flow of air (ca. 200 mL min^−1^) was aspirated across the samples. At constant time intervals, the volatiles evaporating from the cotton surface were adsorbed onto poly(2,6-diphenyl-*p*-phenylene oxide) (Tenax^®^ TA) cartridges, which were then thermally desorbed and the volatiles quantified by gas chromatography (GC) [35,49]. Headspace concentrations measured under realistic application conditions result in relatively large standard deviations because some parameters (such as ambient humidity, temperature and others) are not controlled during the line drying of the cotton sheets and can be quite different from one measurement to another. However, relative differences observed by comparison to the reference samples are typically quite reproducible [35].

**Figure 6:**
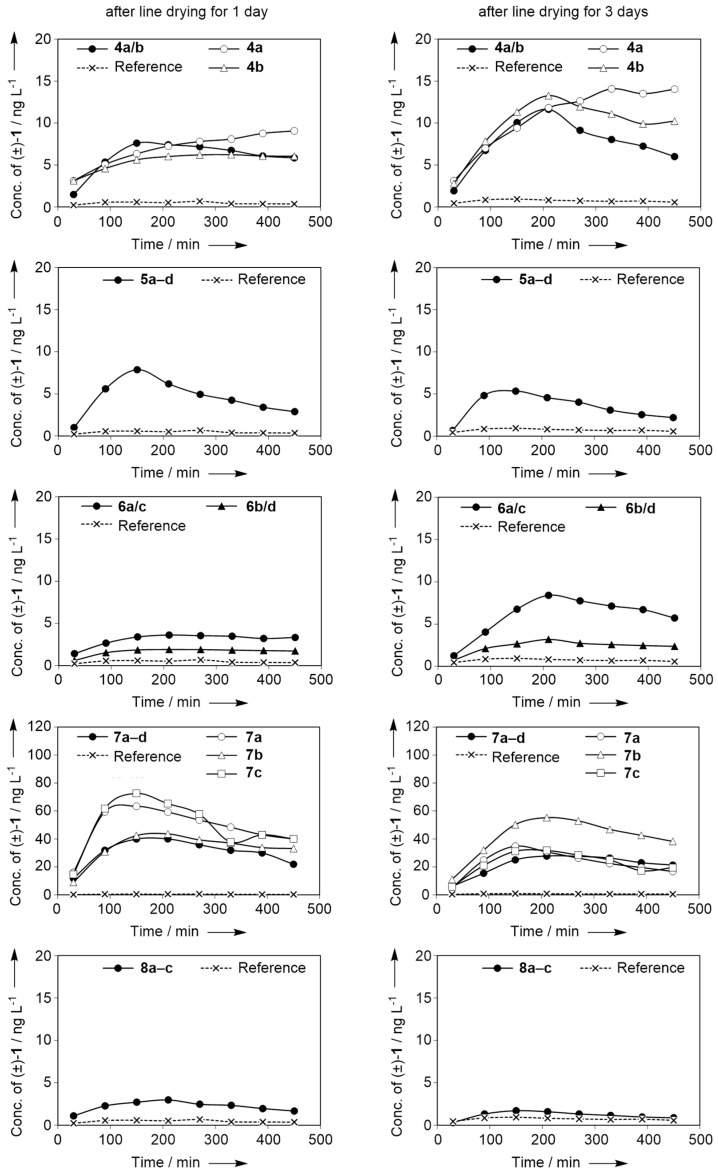
Dynamic headspace concentrations of cyclopentanone derivative (±)-**1** released from profragrances **4**–**8** on dry cotton in a fabric softener application after line drying for 1 day (**left**) or 3 days (**right**). Full symbols correspond to diastereoisomeric mixtures, empty symbols to individual diastereoisomers.

Figure 6 shows a comparison of the dynamic headspace concentrations recorded for the evaporation of unmodified cyclopentanone derivative (±)-**1** (reference) with those released from profragrances **4**–**8** on dry cotton after line drying for 1 day (left) and 3 days (right). Measurements after 1 and 3 days indicate whether the release of the carbonyl compounds is fast (higher headspace concentrations after 1 day than after 3 days) or slow (higher headspace concentrations after 3 days than after 1 day). With respect to the reference sample (equimolar amount of (±)-**1**), higher headspace concentrations were released from the profragrances in all cases. This demonstrates that imidazolidine-4-one-derived profragrances are suitable to generate a long-lasting fragrance perception on cotton in a fabric softening application. Unsurprisingly, the release efficiency of the cyclopentanones depended on the structure of the imidazolidine-4-one derivatives and, in some cases, also on their absolute stereochemistry.

Maximum headspace concentrations of (±)-**1** released from glycinamide-based profragrance **4**, alanine derivative **5**, phenylalanine-derived structure **6** and 2-piperidinecarboxamide-based compound **8** varied between 2 (**8**) and 15 ng L^−1^ (**4**). This corresponded to an average increase of the amount of (±)-**1** evaporating from the cotton surface with respect to the reference by a factor of about 2 (**8**, smallest difference) to 15 (**4**, largest difference) (Factors of increase were estimated from the ratio of the sum of the measured headspace concentration of the fragrance released from the profragrance with respect to the sum of the recorded headspace concentrations of the unmodified reference). Significantly higher headspace concentrations were recorded for *l*-prolinamide-derivative **7**, with headspace concentrations of (±)-**1** well above 20 ng L^−1^ and maximum values reaching up to 80 ng L^−1^, thus corresponding to an increase compared with the reference of between ca. 30 and 100 times. Profragrances **4**–**6** and **8** tendentially released larger amounts of (±)-**1** after 3 days than they did after 1 day, whereas *l*-prolinamide-derivative **7** clearly performed better after line drying for 1 day than it did after 3 days (Please note that the diastereoisomeric mixtures **7a**–**d** and **8a**–**c** used for the headspace analyses did not contain equimolar amounts of the different isomers, but the ratio of isomers obtained from the synthesis before chromatographic separation (**7a**, **7b**, **7c** and **7d** ca. 36%:52%:10%:2% and **8a**, **8b** and **8c** ca. 71%:10%:19%; see Materials and Methods)).

In those cases, where sufficient quantities of purified diastereoisomers were isolated, we also measured the headspace concentrations of (±)-**1** generated for the individual isomers and compared them to those recorded for the crude reaction product consisting of a mixture of all stereoisomers. For glycinamide derivative **4**, similar headspace concentrations were recorded for (5*RS*,6*RS*)-**4a**, (5*RS*,6*SR*)-**4b** and the mixture of **4a**/**b**, thus indicating no significant difference in release efficiency between the different isomers. In the case of *l*-phenylalanine-derivative **6**, we isolated two fractions, each containing a pair of diastereoisomers, notably **6a**/**c** and **6b**/**d**. As shown in Figure 6, the former pair of diastereoisomers released significantly more (±)-**1** than the latter pair did, especially in the measurement after 3 days where about 2.5 times more (±)-**1** was generated from the pair **6a**/**c** than from the pair **6b**/**d**.

The headspace concentrations of (±)-**1** generated from the different diastereoisomers of *l*-prolinamide derivatives **7a**–**d** recorded in the fabric softener application show that the rates of fragrance release depended on the stereochemistry of the profragrances. We observed that, after 1 day of line drying, the highest amount of (±)-**1** was generated from precursors (1*S*,2*S*,7a′*S*)-**7a** and (1*R*,2*R*,7a′*S*)-**7c**, followed by (1*S*,2*R*,7a′*S*)-**7b**, the performance of which was close to that of the mixture of isomers **7a**–**d** (Please note that the diastereoisomeric mixtures **7a**–**d** and **8a**–**c** used for the headspace analyses did not contain equimolar amounts of the different isomers, but the ratio of isomers obtained from the synthesis before chromatographic separation (**7a**, **7b**, **7c** and **7d** ca. 36%:52%:10%:2% and **8a**, **8b** and **8c** ca. 71%:10%:19%; see Materials and Methods)). After 3 days, **7b** released slightly higher amounts of (±)-**1** than **7a** and **7c** did, both performing about equally well as the diastereoisomeric mixture of **7a**–**d**.

In contrast to the results of equilibration studies recorded earlier in methanol, the fragrance release on the cotton surface occurred under non-equilibrium conditions. The constant evaporation of the fragrance during the headspace sampling irreversibly shifted the equilibrium towards the hydrolysis of the profragrances to form *l*-prolinamide and (±)-**1**.

Our headspace data indicated that the release of (±)-**1** on dry cotton was faster from **7a** and **7c** than from **7b**, which correlated with our previous observation from the NMR measurements. We observed that **7a** rapidly released *l*-prolinamide, followed by the energetically higher isomer **7c**, whereas **7b** formed *l*-prolinamide quite slowly (see inserts in Scheme 7), which might explain the observed differences for the rates of fragrance release in application.

**Figure 7:**
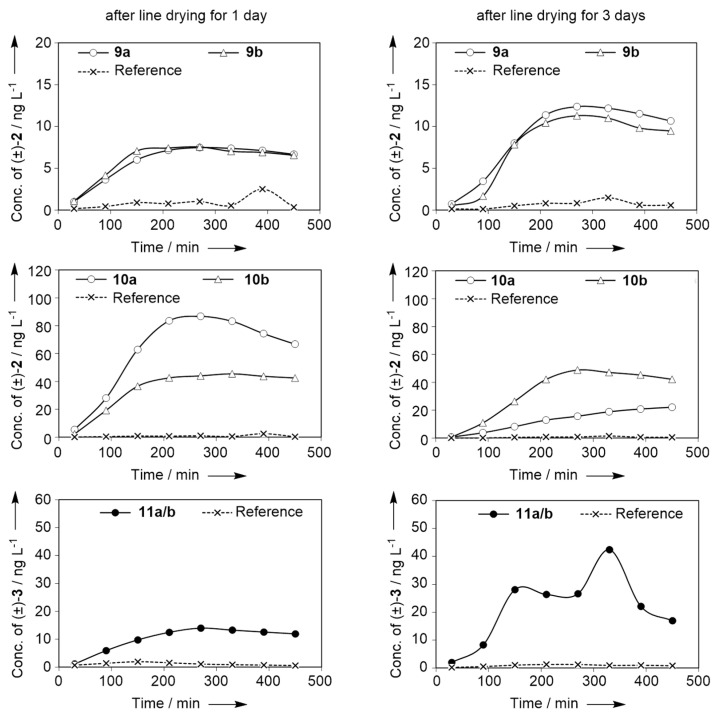
Dynamic headspace concentrations of cyclopentanone derivatives (±)-**2** and (±)-**3** released from profragrances **9**–**11** on dry cotton in a fabric softener application after line drying for 1 day (**left**) or 3 days (**right**). Full symbols correspond to diastereoisomeric mixtures, empty symbols to individual diastereoisomers.

Using the same method, we then investigated the release of cyclopentanone (±)-**2** from glycinamide-derived profragrances (5*RS*,6*RS*)-**9a** and (5*RS*,6*SR*)-**9b** and from *l*-prolinamide-based profragrances (1*S*,2*S*,7a′*S*)-**10a** and (1*S*,2*R*,7a′*S*)-**10b**, as well as of cyclopentanone (±)-**3** from glycinamide-derived precursor **11a**/**b**. The headspace concentrations of the fragrances released from the different precursors were compared against an equimolar amount of the corresponding unmodified reference fragrance. The recorded headspace concentrations on dry cotton after line drying for 1 day and 3 days are shown in Figure 7.

As previously observed for the release of (±)-**1** from **4a** and **4b**, the two diastereoisomers **9a** and **9b** released comparable quantities of (±)-**2** (around 10 ng L^−1^), independent of their individual stereochemistry. Larger headspace concentrations (with values slightly above 10 ng L^−1^) were obtained after 3 days than after 1 day (concentrations below 10 ng L^−1^), indicating that the release of (±)-**2** was rather slow. Glycinamide-based profragrance **11a**/**b** released around 10 ng L^−1^ of (±)-**3** and up to about 30 ng L^−1^ 3 days (Figure 7).

Again, as described earlier for the release of (±)-**1** from **7a** and **7b**, *l*-prolinamide derivatives **10a** and **10b** generated significant amounts of (±)-**2** (up to 80 ng L^−1^). More (±)-**2** was released after 1 day than after 3 days, indicating a rather rapid release of the cyclopentanone fragrance. The amount of fragrance released depended on the stereochemistry of the profragrances. After 1 day, (1*S*,2*S*,7a′*S*)-**10a** released almost twice as much of (±)-**2** than (1*S*,2*R*,7a′*S*)-**10b**, whereas after 3 days, the trend was inverted, with **10b** releasing more than two times more fragrance than **10a** (Figure 7). Imidazolidin-4-one derivative **10a** thus hydrolyses more rapidly than its diastereoisomer **10b**. These findings correlated well with our previous observations for the hydrolysis of (1*S*,2*S*,7a′*S*)-**7a** and (1*S*,2*R*,7a′*S*)-**7b** (see Figure 6).

Studying the fragrance release from individual diastereoisomers is mainly of academic interest to understand the mechanisms involved in the synthesis of the profragrances and their fragrance release. Cost being an important parameter for the selection of fragrance delivery systems in consumer products [12], the separation of individual isomers would be prohibitive for any practical application. However, because of the formation of thermodynamic equilibria, the separation of diastereoisomers is not necessary, as, in any case, the same mixture of compounds is expected to be reached after sufficient equilibration times.

## 3. Materials and Methods

### 3.1. Synthesis and Characterisation of Imidazolidin-4-One Derivatives ***4***–***11*** [39]

#### 3.1.1. Synthesis of 6-pentyl-1,4-diazaspiro[4.4]nonan-2-one (**4a**/**b**)

Triethylamine (TEA, 20 mL, 14.52 g, 143 mmol) and (±)-**1** (10.03 g, 65 mmol) were added to a suspension of glycinamide hydrochloride (14.66 g, 130 mmol) in methanol (250 mL). The reaction mixture was heated under reflux for 120 h (5 d). After cooling to room temperature, the solvent was evaporated under reduced pressure. Ethyl acetate (150 mL) and water (150 mL) were added, and the biphasic mixture was stirred for 15 min. The phases were separated and the aqueous phase re-extracted with ethyl acetate (150 mL). The combined organic phases were washed with a saturated aqueous solution of NaCl (2 × 100 mL), dried (Na_2_SO_4_), filtered and concentrated under reduced pressure. Bulb-to-bulb distillation (100 °C, 0.8 mbar) to remove remaining (±)-**1** afforded 10.37 g of the crude compound containing **4a**, **4b** and (±)-**12** in a ratio of ca. 34%:35%:31%, which might be used as such for application.

Column chromatography (SiO_2_, ethyl acetate/ethanol 3:1) of the crude compound (1.00 g) afforded 0.63 g (48%) of **4a**/**b** (ca. 1:1), which represents a typical sample used for headspace analysis.

Column chromatography (SiO_2_, ethyl acetate, then ethyl acetate/ethanol 9:1) of the crude compound (2.00 g) afforded 0.36 g (14%) of **4a**, 0.82 g (31%) of a mixed fraction containing **4a**/**b** ca. 37%:64% and 0.09 g (3%) of **4b**.

(5*RS*,6*RS*)-**4a**: ^1^H NMR (500 MHz, CDCl_3_), ppm: *δ* = 6.62 (br. s, 1 H), 3.45 (dd, J = 41.4, 25.0 Hz, 2 H), 1.96–1.60 (m, 7 H), 1.55–1.15 (m, 9 H), 0.88 (t, J = 6.9 Hz, 3 H). ^13^C NMR (125.8 MHz, CDCl_3_), ppm: *δ* = 176.72 (s), 85.16 (s), 49.69 (t), 48.74 (d), 39.35 (t), 32.16 (t), 28.39 (t), 28.17 (t), 28.08 (t), 22.58 (t), 19.79 (t), 14.05 (q). IR (neat), cm^–1^: 3293 m, 3180 m (br.), 3093 m (br.), 2956 m, 2922 m, 2871 w, 2855 m, 1819 w, 1698 s, 1685 m, 1557 w, 1457 m, 1440 m, 1377 w, 1350 m, 1327 m, 1226 w, 1206 w, 1181 w, 1147 w, 1116 m, 1085 w, 1063 w, 1033 w, 1013 w, 981 w, 913 m, 873 w, 838 m, 818 m, 725 m, 654 m. HRMS (Q-Exactive) (*m*/*z*): calcd. for C_12_H_23_N_2_O^+^ [M + H]^+^ 211.18049, found 211.18050.

(5*RS*,6*SR*)-**4b**: ^1^H NMR (500 MHz, CDCl_3_), ppm: *δ* = 7.08 (br. S, 1 H), 3.49 (dd, J = 16.3, 2.6 Hz, 2 H), 2.08–1.62 (m, 7 H), 1.50–1.06 (m, 9 H), 0.88 (t, J = 6.9 Hz, 3 H). ^13^C NMR (125.8 MHz, CDCl_3_), ppm: *δ* = 176.62 (s), 86.12 (s), 49.21 (t), 48.97 (d), 39.56 (t), 32.08 (t), 29.69 (t), 29.47 (t), 27.96 (t), 22.60 (t), 20.54 (t), 14.04 (q). IR (neat), cm^–1^: 3308 w, 3195 m (br.), 3087 m (br.), 2956 m, 2926 m, 2871 w, 2857 m, 1689 s, 1562 w, 1436 m, 1377 w, 1355 m, 1324 m, 1201 w, 1182 w, 1150 w, 1114 m, 1093 w, 983 w, 753 m, 726 m. HRMS (Q-Exactive) (*m*/*z*): calcd. for C_12_H_23_N_2_O^+^ [M + H]^+^ 211.18049, found 211.18050.

#### 3.1.2. Synthesis of (3S)-3-methyl-6-pentyl-1,4-diazaspiro[4.4]nonan-2-one (**5a**–**d**)

TEA (15.3 mL, 110 mmol) and (±)-**1** (7.71 g, 50 mmol) were added to a solution of *l*-alaninamide hydrochloride (12.84 g, 100 mmol) in methanol (200 mL). The reaction mixture was heated under reflux for 5 d. After cooling to room temperature, the solvent was evaporated under reduced pressure. Water (100 mL) was added and the mixture extracted with ethyl acetate (100 mL). After phase separation, the aqueous phase was re-extracted with ethyl acetate (100 mL). The combined organic phases were washed with a saturated aqueous solution of NaCl (100 mL), dried (Na_2_SO_4_), filtered and concentrated under reduced pressure. Bulb-to-bulb distillation (110 °C, 0.9 mbar) to remove remaining (±)-**1** afforded 9.83 g of the crude compound as a viscous yellow oil, containing **5a**–**5d** and the corresponding imine in a ratio of ca. 24%:20%:26%:26%:4%, which might be used as such for application.

Column chromatography (SiO_2_, ethyl acetate/ethanol 3:1) of the crude compound (1.01 g) afforded 0.97 g (84%) of **5a**–**5d** in a ratio of ca. 26%:21%:27%:26%, which represents a typical sample used for headspace analysis.

Column chromatography (SiO_2_, *n*-heptane/ethyl acetate 2:1, then 1:1, then ethyl acetate) of the crude compound (2.00 g) afforded 0.22 g (10%) of **5a** as a colourless viscous oil, 0.19 g (8%) of a mixture of **5a** and **5b** in a ratio of 74%:26% as white crystals, 1.16 g (51%) of mixed fractions containing at least three isomers, and 0.21 g (9%) of a mixture of **5c** and **5d** in a ratio of 18%:82% as a colourless viscous oil. Column chromatography (SiO_2_, *n*-heptane/ethyl acetate 1:1) of several of the mixed fractions afforded 0.06 g of **5c** and **5d** in a ratio of 64%:36% and 0.04 g of **5d** (containing ca. 7% of **5c**).

(3*S*,5*R*,6*R* or 3*S*,5*S*,6*S*)-**5a**: ^1^H NMR (600 MHz, CDCl_3_), ppm: *δ* = 6.79 (br. s, 1 H), 3.53 (q, J = 6.9 Hz, 1 H), 2.08–1.99 (m, 1 H), 1.92–1.83 (m, 1 H), 1.78–1.59 (m, 5 H), 1.53–1.15 (m, 9 H), 1.35 (d, J = 6.6 Hz, 3 H), 0.87 (t, J = 6.9 Hz, 3 H). ^13^C NMR (150.9 MHz, CDCl_3_), ppm: *δ* = 178.68 (s), 83.00 (s), 55.79 (d), 49.90 (d), 40.99 (t), 32.21 (t), 28.46 (t), 28.40 (t), 28.11 (t), 22.59 (t), 19.76 (t), 18.52 (q), 14.06 (q). IR (neat), cm^–1^: 3285 m, 3204 m (br.), 3106 w, 2955 m, 2924 m, 2874 w, 2857 m, 1693 s, 1464 m, 1457 w, 1440 m, 1424 m, 1375 m, 1339 m, 1327 w, 1307 w, 1281 w, 1201 w, 1145 w, 1125 m, 1105 m, 1076 w, 1057 m, 1018 w, 932 w, 905 w, 860 w, 828 m, 807 m, 729 m. HRMS (Q-Exactive) (*m*/*z*): calcd. for C_13_H_25_N_2_O^+^ [M + H]^+^ 225.19614, found 225.19617.

(3*S*,5*S*,6*S* or 3*S*,5*R*,6*R*)-**5b**: ^1^H NMR (600 MHz, CDCl_3_), minor isomer in a mixture with **5a** (26:74), ppm: *δ* = 7.02 (br. s, 1 H), 3.61 (q, J = 6.8 Hz, 1 H), 2.09–1.92 (m, 3 H), 1.92–1.59 (m, 4 H), 1.52–1.16 (m, 8 H), 1.34 (d, J = 6.9 Hz, 3 H), 1.15–1.06 (m, 1 H), 0.89 (t, J = 6.9 Hz, 3 H). ^13^C NMR (150.9 MHz, CDCl_3_), minor isomer in a mixture with **5a** (26:74), ppm: *δ* = 178.78 (s), 82.94 (s), 54.23 (d), 46.14 (d), 38.49 (t), 32.11 (t), 28.47 (t), 28.22 (t), 27.93 (t), 22.57 (t), 19.73 (t), 17.01 (q), 14.05 (q). HRMS (Q-Exactive) (*m*/*z*): calcd. for C_13_H_25_N_2_O^+^ [M + H]^+^ 225.19614, found 225.19614 and 225.19618.

(3*S*,5*S*,6*R* or 3*S*,5*R*,6*S*)-**5c**: ^1^H NMR (500 MHz, CDCl_3_), major isomer in a mixture with **5d** (64:36), ppm: *δ* = 7.50 (br. *s*, 1 H), 3.64 (q, J = 6.9 Hz, 1 H), 2.06–1.58 (m, 7 H), 1.49–1.16 (m, 8 H), 1.34 (d, J = 6.7 Hz, 3 H), 1.15–1.04 (m, 1 H), 0.87 (t, J = 6.9 Hz, 3 H). ^13^C NMR (125.8 MHz, CDCl_3_), major isomer in a mixture with **5d** (64:36), ppm: *δ* = 178.72 (s), 84.01 (s), 54.99 (d), 49.40 (d), 40.67 (t), 32.14 (t), 29.55 (t), 29.07 (t), 28.00 (t), 22.62 (t), 20.29 (t), 18.67 (q), 14.05 (q). HRMS (Q-Exactive) (*m*/*z*): calcd. for C_13_H_25_N_2_O^+^ [M + H]^+^ 225.19614, found 225.19612 and 225.19611.

(3*S*,5*R*,6*S* or 3*S*,5*S*,6*R*)-**5d**: ^1^H NMR (500 MHz, CDCl_3_), ppm: *δ* = 7.78 (br. s, 1 H), 3.59 (q, J = 6.9 Hz, 1 H), 2.05–1.96 (m, 1 H), 1.94–1.84 (m, 2 H), 1.84–1.76 (m, 1 H), 1.73–1.58 (m, 3 H), 1.48–1.18 (m, 8 H), 1.34 (d, J = 7.1 Hz, 3 H), 1.15–1.04 (m, 1 H), 0.88 (t, J = 6.9 Hz, 3 H). ^13^C NMR (125.8 MHz, CDCl_3_), ppm: *δ* = 178.65 (s), 83.83 (s), 54.34 (d), 48.33 (d), 39.98 (t), 32.14 (t), 29.64 (t), 29.60 (t), 27.99 (t), 22.62 (t), 20.91 (t), 17.55 (q), 14.06 (q). IR (neat), cm^–1^: 3195 m (br.), 3084 w (br.), 2957 m, 2926 m, 2872 w, 2857 m, 2239 w, 1694 s, 1453 m, 1367 m, 1353 m, 1325 m, 1238 w, 1213 w, 1189 w, 1136 w, 1100 w, 1060 w, 1045 w, 1018 w, 946 w, 921 w, 909 w, 765 m, 731 s. HRMS (Q-Exactive) (*m*/*z*): calcd. for C_13_H_25_N_2_O^+^ [M + H]^+^ 225.19614, found 225.19614.

#### 3.1.3. Synthesis of (3S)-3-benzyl-6-pentyl-1,4-diazaspiro[4.4]nonan-2-one (**6a**–**d**)

TEA (7.7 mL, 55 mmol) and (±)-**1** (3.86 g, 25 mmol) were added to a solution of *l*-phenylalaninamide hydrochloride (10.03 g, 50 mmol) in methanol (100 mL). The reaction mixture was heated under reflux for 5 d. After cooling to room temperature, the solvent was evaporated under reduced pressure. Water (100 mL) was added and the mixture extracted with ethyl acetate (100 mL). After phase separation, the aqueous phase was re-extracted with ethyl acetate (100 mL). The combined organic phases were washed with a saturated aqueous solution of NaCl (100 mL), dried (Na_2_SO_4_), filtered and concentrated under reduced pressure. Bulb-to-bulb distillation (110 °C, 0.9 mbar) to remove remaining (±)-**1** afforded 8.50 g of the crude compound as a viscous yellow oil, containing **6a**–**d** and the corresponding imine in a ratio of ca. 21%:20%:22%:27%:10%, which might be used as such for application. Column chromatography (SiO_2_, ethyl acetate/ethanol 3:1) of the crude compound (1.10 g) afforded 1.01 g of **6a**–**d** in a ratio of ca. 23%:24%:24%:29%, which represents a typical sample used for headspace analysis.

Column chromatography (SiO_2_, *n*-heptane/ethyl acetate 1:1, then 1:2) of the crude compound (2.00 g) afforded 0.76 g (43%) of **6a** and **6c** in a ratio of 50%:50% (containing small amounts of (±)-**1**) and 0.86 g (49%) of **6b** and **6d** in a ratio of 45%:55%.

(3*S*,5*R*,6*R* or 3*S*,5*S*,6*S*)-**6a**: ^1^H NMR (500 MHz, CDCl_3_), ppm: *δ* = 7.33–7.21 (m, 5 H), 6.92 (br. s, 1 H), 3.79 (t, J = 5.3 Hz, 1 H), 3.12–3.01 (m, 2 H), 1.88–1.72 (m, 2 H), 1.72–1.50 (m, 3 H), 1.50–1.38 (m, 1 H), 1.38–1.03 (m, 9 H), 0.87 (t, J = 7.1 Hz, 3 H), one signal for NH not assigned. ^13^C NMR (125.8 MHz, CDCl_3_), ppm: *δ* = 177.07 (s), 136.88 (s), 129.66 (d), 128.63 (d), 126.84 (d), 83.16 (s), 60.81 (d), 49.77 (d), 40.48 (t), 37.48 (t), 32.21 (t), 28.35 (t), 28.25 (t), 28.07 (t), 22.61 (t), 19.63 (t), 14.07 (q). (3*S*,5*S*,6*R* or 3*S*,5*R*,6*S*)-**6c**: ^1^H NMR (500 MHz, CDCl_3_), ppm: *δ* = 7.35 (br. s 1 H), 7.33–7.21 (m, 5 H), 3.88 (dd, J = 7.3, 4.2 Hz, 1 H), 3.12–3.01 (m, 1 H), 2.95 (dd, J = 13.8, 7.3 Hz, 1 H), 2.01–1.90 (m, 1 H), 1.88–1.72 (m, 1 H), 1.72–1.50 (m, 4 H), 1.50–1.38 (m, 2 H), 1.38–1.03 (m, 7 H), 0.86 (t, J = 7.1 Hz, 3 H), one signal for NH not assigned. ^13^C NMR (125.8 MHz, CDCl_3_), ppm: *δ* = 176.86 (s), 137.36 (s), 129.64 (d), 128.56 (d), 126.76 (d), 84.22 (s), 60.35 (d), 49.65 (d), 40.54 (t), 38.28 (t), 32.15 (t), 29.31 (t), 28.94 (t), 28.05 (t), 22.64 (t), 20.04 (t), 14.04 (q). HRMS (Q-Exactive) (*m*/*z*): calcd. for C_19_H_29_N_2_O^+^ [M + H]^+^ 301.22744, found 301.22748 and 301.22733.

(3*S*,5*S*,6*S* or 3*S*,5*R*,6*R*)-**6b**: ^1^H NMR (500 MHz, CDCl_3_, minor isomer), ppm: *δ* = 7.34–7.21 (m, 5 H), 6.65 (br. s, 1 H), 3.81 (q, J = 5.1 Hz, 1 H), 3.20 (dd, J = 14.1, 5.1 Hz, 1 H), 3.04 (dd, J = 12.2, 5.1 Hz, 1 H), 1.99–1.82 (m, 2 H), 1.74–1.58 (m, 4 H), 1.34–0.96 (m, 7 H), 0.91–0.70 (m, 1 H), 087 (t, J = 7.2 Hz, 3 H), 0.64–0.53 (m, 1 H), one signal for NH not assigned. ^13^C NMR (125.8 MHz, CDCl_3_, minor isomer), ppm: *δ* = 177.15 (s), 136.66 (s), 129.94 (d), 128.71 (d), 126.99 (d), 82.80 (s), 59.01 (d), 46.44 (d), 38.69 (t), 35.77 (t), 32.04 (t), 28.40 (t), 27.91 (t), 27.36 (t), 22.45 (t), 19.67 (t), 14.04 (q). (3*S*,5*R*,6*S* or 3*S*,5*S*,6*R*)-**6d**: ^1^H NMR (500 MHz, CDCl_3_, major isomer), ppm: *δ* = 7.34–7.21 (m, 5 H), 7.14 (br. s, 1 H), 3.81 (q, J = 5.1 Hz, 1 H), 3.12 (dd, J = 14.1, 5.4 Hz, 1 H), 3.06 (dd, J = 14.1, 4.8 Hz, 1 H), 1.99–1.74 (m, 3 H), 1.74–1.58 (m, 2 H), 1.50–1.41 (m, 1 H), 1.34–0.96 (m, 7 H), 0.91–0.70 (m, 2 H), 0.87 (t, J = 7.4 Hz, 3 H), one signal for NH not assigned. ^13^C NMR (125.8 MHz, CDCl_3_, major isomer), ppm: *δ* = 176.97 (s), 136.74 (s), 129.79 (d), 128.67 (d), 126.96 (d), 83.67 (s), 59.30 (d), 47.89 (d), 40.39 (t), 36.35 (t), 31.86 (t), 29.64 (t), 28.99 (t), 27.78 (t), 22.50 (t), 20.84 (t), 14.06 (q). HRMS (Q-Exactive) (*m*/*z*): calcd. for C_19_H_29_N_2_O^+^ [M + H]^+^ 301.22744, found 301.22739 and 301.22748.

#### 3.1.4. Synthesis of (7a′S)-2-pentyltetrahydrospiro[cyclopentane-1,3′-pyrrolo[1,2-c]imidazol]-1′(2′H)-one (**7a**–**d**)

TEA (20 mL, 14.52 g, 143 mmol) and (±)-**1** (10.00 g, 65 mmol) were added to a solution of *l*-prolinamide hydrochloride (20.61 g, 130 mmol) in methanol (250 mL). The reaction mixture was heated under reflux for 5 d. After cooling to room temperature, the solvent was evaporated under reduced pressure. Ethyl acetate (100 mL) and water (100 mL) were added, and the biphasic mixture was stirred for 15 min. The phases were separated and the aqueous phase re-extracted with ethyl acetate (100 mL). The combined organic phases were washed with a saturated aqueous solution of NaCl (100 mL), dried (Na_2_SO_4_), filtered and concentrated under reduced pressure. Bulb-to-bulb distillation (110 °C, 0.9 mbar) to remove remaining (±)-**1** afforded 13.17 g (81%) of the crude compound containing **7a**, **7b**, **7c** and **7d** in a ratio of ca. 36%:52%:10%:2%, which represents a typical sample used for headspace analysis.

Column chromatography (SiO_2_, ethyl acetate/*n*-heptane 2:1, then ethyl acetate) of the crude compound (2.00 g) afforded 0.27 g (11%) of **7a**, 1.07 g (43%) of a mixed fraction containing **7a/b** in a ratio of ca. 38%:62%, 0.41 g (17%) of **7b** and 0.24 g (10%) of **7c**. Due to the small amount formed in the reaction, diastereoisomer **7d** was not isolated.

(1*S*,2*S*,7a′*S*)-**7a**: ^1^H NMR (500 MHz, CDCl_3_), ppm: *δ* = 7.27 (br. s, 1 H), 3.79 (dd, J = 9.6, 5.4 Hz, 1 H), 2.95–2.88 (m, 1 H), 2.75–2.65 (m, 1 H), 2.15–2.03 (m, 2 H), 1.95–1.63 (m, 8 H), 1.61–1.51 (m, 1 H), 1.51–1.41 (m, 1 H), 1.39–1.12 (m, 7 H), 0.87 (t, J = 6.9 Hz, 3 H). ^13^C NMR (125.8 MHz, CDCl_3_), ppm: *δ* = 179.13 (s), 87.10 (s), 65.11 (d), 51.78 (d), 50.35 (t), 32.24 (t), 32.15 (t), 29.55 (t), 28.00 (t), 27.59 (t), 27.06 (t), 25.62 (t), 22.68 (t), 19.84 (t), 14.09 (q). IR (neat), cm^–1^: 3192 m (br.), 3076 w (br.), 2955 m, 2925 m, 2871 w, 2858 m, 1694 s, 1456 m, 1446 m, 1429 w, 1377 w, 1361 m, 1338 m, 1308 w, 1287 w, 1269 w, 1253 w, 1198 m, 1118 m, 1074 m, 1045 w, 1018 w, 969 w, 941 w, 922 w, 908 w, 888 w, 797 m, 768 w, 726 m, 691 w. HRMS (Q-Exactive) (*m*/*z*): calcd. for C_15_H_27_N_2_O^+^ [M + H]^+^ 251.21179, found 251.21181.

(1*S*,2*R*,7a′*S*)-**7b**: ^1^H NMR (500 MHz, CDCl_3_), ppm: *δ* = 7.83 (br. s, 1 H), 3.93 (dd, J = 9.3, 5.4 Hz, 1 H), 3.05–2.99 (m, 1 H), 2.77–2.67 (m, 1 H), 2.19–2.01 (m, 2 H), 1.99–1.87 (m, 3 H), 1.86–1.67 (m, 5 H), 1.57–1.48 (m, 1 H), 1.38–1.15 (m, 7 H), 1.13–1.03 (m, 1 H), 0.86 (t, J = 7.1 Hz, 3 H). ^13^C NMR (125.8 MHz, CDCl_3_), ppm: *δ* = 179.33 (s), 87.73 (s), 64.83 (d), 50.95 (d), 50.61 (t), 33.24 (t), 32.26 (t), 29.84 (t), 27.94 (t), 27.65 (t), 27.18 (t), 25.54 (t), 22.70 (t), 20.13 (t), 14.07 (q). IR (neat), cm^–1^: 3179 m (br.), 3075 w (br.), 2955 m, 2926 m, 2872 w, 2857 m, 1694 s, 1448 m, 1433 m, 1377 m, 1362 m, 1339 m, 1318 w, 1300 w, 1272 w, 1250 w, 1238 w, 1205 w, 1161 w, 1138 m, 1101 w, 1038 w, 1017 w, 994 w, 941 w, 897 w, 866 w, 797 m, 726 m, 706 m. HRMS (Q-Exactive) (*m*/*z*): calcd. for C_15_H_27_N_2_O^+^ [M + H]^+^ 251.21179, found 251.21176.

(1*R*,2*R*,7a′*S*)-**7c**: ^1^H NMR (500 MHz, CDCl_3_), ppm: *δ* = 7.00 (br. s, 1 H), 3.84 (dd, J = 9.6, 4.7 Hz, 1 H), 3.02–2.95 (m, 1 H), 2.64–2.55 (m, 1 H), 2.19–2.05 (m, 3 H), 2.05–1.93 (m, 2 H), 1.90–1.73 (m, 2 H), 1.73–1.58 (m, 3 H), 1.50–1.20 (m, 8 H), 1.19–1.08 (m, 1 H), 0.89 (t, J = 7.0 Hz, 3 H). ^13^C NMR (125.8 MHz, CDCl_3_), ppm: *δ* = 179.33 (s), 86.69 (s), 63.72 (d), 48.17 (t), 42.79 (d), 40.30 (t), 31.88 (t), 31.46 (t), 30.78 (t), 27.90 (t), 25.71 (t), 25.32 (t), 22.61 (t), 21.69 (t), 14.06 (q). IR (neat), cm^–1^: 3188 m (br.), 3077 w (br.), 2954 m, 2928 m, 2858 m, 1695 s, 1464 m, 1449 m, 1421 m, 1361 m, 1336 w, 1317 w, 1297 w, 1253 w, 1237 w, 1201 w, 1166 m, 1141 w, 1098 m, 1016 w, 1001 w, 941 w, 905 w, 890 w, 873 w, 807 m, 768 m, 725 m, 657 w. HRMS (Q-Exactive) (*m*/*z*): calcd. for C_15_H_27_N_2_O^+^ [M + H]^+^ 251.21179, found 251.21185.

#### 3.1.5. Synthesis of 2-pentyltetrahydro-2′H-spiro[cyclopentane-1,3′-imidazo[1,5-a]pyridin]-1′(5′H)-one (**8a**–**d**)

TEA (0.85 mL, 6 mmol) and (±)-**1** (4.63 g, 30 mmol) were added to a solution of 2-piperidinecarboxamide (8.09 g, 60 mmol) in methanol (50 mL). The reaction mixture was heated under reflux for 45 h and, after cooling to room temperature, concentrated. The residue was taken up in ethyl acetate (150 mL) and treated with water (50 mL). The aqueous phase was extracted with ethyl acetate (150 mL), and the combined organic phases were washed with water (50 mL) and a saturated aqueous solution of NaCl (50 mL), dried (Na_2_SO_4_), filtered and concentrated. Bulb-to-bulb distillation (130 °C, 0.4 mbar) to remove the remaining (±)-**1** yielded 3.14 g (40%) of the crude compound as a brownish yellow oil, containing diastereoisomers (±)-**8a**–**c** in a ratio of ca. 71%:10%:19%, which represents a typical sample used for headspace analysis.

Column chromatography (SiO_2_, toluene/ethyl acetate 4:1, then 3:2) of the crude compound (2.00 g) afforded 0.87 g (17%) of **8a** as a yellow solid, 0.27 g (3%) of a mixture of (±)-**8a** and (±)-**8b** in a ratio of ca. 44%:56% as a slightly yellow oil and 0.29 g (6%) of (±)-**8c** as a brown solid.

(1*RS*,2*RS*,8a′*SR*)-**8a**: ^1^H NMR (600 MHz, CDCl_3_), ppm: *δ* = 6.93 (br. s, 1 H), 2.88–2.81 (m, 2 H), 2.27 (dt, J = 11.0, 2.3 Hz, 1 H), 2.02–1.82 (m, 4 H), 1.77–1.43 (m, 7 H), 1.39–1.11 (m, 9 H), 1.03–0.94 (m, 1 H), 0.88 (t, J = 6.9 Hz, 3 H). ^13^C NMR (151.0 MHz, CDCl_3_), ppm: *δ* = 174.97 (s), 86.01 (s), 61.94 (d), 44.48 (t), 43.09 (d), 32.09 (t), 29.26 (t), 29.00 (t), 28.67 (t), 28.15 (t), 26.47 (t), 25.57 (t), 24.46 (t), 22.63 (t), 20.52 (t), 14.10 (q). IR (neat), cm^–1^: 3371 w, 3176 m (br.), 3092 m, 3070 m, 2954 m, 2931 m, 2875 m, 2855 m, 2825 w, 2734 w, 2163 w, 1739 w, 1700 s, 1653 w, 1469 w, 1455 m, 1439 m, 1397 m, 1362 m, 1348 m, 1326 m, 1306 m, 1288 m, 1270 w, 1255 m, 1237 w, 1220 m, 1209 m, 1196 w, 1177 m, 1151 m, 1128 w, 1078 m, 1068 w, 1053 m, 1029 w, 994 w, 978 w, 941 m, 921 w, 903 w, 874 w, 845 m, 766 m, 732 m, 684 m. HRMS (Q-Exactive) (*m*/*z*): calcd. for C_16_H_29_N_2_O^+^ [M + H]^+^ 265.22744, found 265.22742.

(1*SR*,2*SR*,8a′*SR*)-**8b**: ^1^H NMR (600 MHz, CDCl_3_), major isomer in a mixture with **8a** (56:44), ppm: *δ* = 6.45 (br. s, 1 H), 2.89–2.81 (m, 1 H), 2.81–2.75 (m, 1 H), 2.38 (dt, J = 10.9, 2.7 Hz, 1 H), 2.24–2.14 (m, 1 H), 2.03–1.82 (m, 3 H), 1.82–1.10 (m, 17 H), 0.88 (t, J = 6.9 Hz, 3 H). ^13^C NMR (151.0 MHz, CDCl_3_), major isomer in a mixture with **8a** (56:44), ppm: *δ* = 174.91 (s), 85.04 (s), 62.21 (d), 48.20 (d), 47.35 (t), 32.29 (t), 30.90 (t), 30.28 (t), 29.27 (t), 28.22 (t), 26.36 (t), 25.20 (t), 24.15 (t), 22.67 (t), 21.46 (t), 14.13 (q). HRMS (Q-Exactive) (*m*/*z*): calcd. for C_16_H_29_N_2_O^+^ [M + H]^+^ 265.22744, found 265.22742 and 265.22739.

(1*RS*,2*SR*,8a′*SR*)-**8c**: ^1^H NMR (600 MHz, CDCl_3_), ppm: *δ* = 6.94 (br. s, 1 H), 3.31–3.24 (m, 1 H), 3.00–2.93 (m, 1 H), 2.52 (dt, J = 11.6, 2.7 Hz, 1 H), 2.01–1.83 (m, 5 H), 1.77–1.11 (m, 16 H), 0.88 (t, J = 6.9 Hz, 3 H). ^13^C NMR (151.0 MHz, CDCl_3_), ppm: *δ* = 175.51 (s), 86.05 (s), 61.71 (d), 43.61 (t), 43.33 (d), 37.17 (t), 32.13 (t), 30.36 (t), 29.03 (t), 28.01 (t), 27.16 (t), 25.52 (t), 24.24 (t), 22.66 (t), 19.94 (t), 14.06 (q). IR (neat), cm^–1^: 3184 m (br.), 3087 m (br.), 2924 m, 2873 m, 2855 m, 2814 w, 2760 w, 2324 w, 2284 w, 2161 w, 2051 w, 1980 w, 1699 s, 1590 w, 1575 w, 1439 m, 1395 w, 1379 w, 1362 m, 1343 m, 1332 w, 1304 m, 1283 m, 1255 w, 1222 m, 1203 m, 1180 w, 1157 w, 1146 m, 1129 m, 1098 w, 1072 w, 1050 w, 1032 w, 946 w, 934 w, 919 w, 876 w, 842 w, 781 m, 732 m, 680 m. HRMS (Q-Exactive) (*m*/*z*): calcd. for C_16_H_29_N_2_O^+^ [M + H]^+^ 265.22744, found 265.22742.

#### 3.1.6. Synthesis of 6-(hex-5-enyl)-1,4-diazaspiro[4.4]nonan-2-one (**9a**/**b**)

In a Soxhlet extractor with molecular sieves (4 Å), a mixture of 2-(5-hexenyl)cyclopentan-1-one ((±)-**2**, 1.73 g, 10 mmol), TEA (1.5 mL, 11 mmol) and glycinamide hydrochloride (1.13 g, 10 mmol) in methanol (125 mL) was heated under reflux for several days. After cooling to room temperature, the solvent was evaporated under reduced pressure and the mixture taken up in ethyl acetate (50 mL) and water (25 mL). After separation, the aqueous phase was re-extracted with ethyl acetate (50 mL) and the organic phases washed with a saturated aqueous solution of NaCl (25 mL). The combined organic phases were dried (Na_2_SO_4_), filtered and concentrated under reduced pressure to give 1.85 g (83%) of the crude product, which represents a typical sample used for headspace analysis.

Column chromatography (SiO_2_, ethyl acetate) afforded 0.15 g of **9a**; further elution (ethyl acetate/ethanol) yielded 0.24 g of a mixture of isomers and 0.19 g of **9b** as brownish-yellow, viscous oils. The stereochemistry of the two diastereoisomers was assigned in analogy to the chemical shifts recorded in the ^13^C NMR spectra of **4a**/**b** (see earlier).

(5*RS*,6*RS*)-**9a**: ^1^H NMR (500 MHz, CDCl_3_), ppm: *δ* = 6.89 (br. s, 1 H), 5.85–5.74 (m, 1 H), 5.03–4.91 (m, 2 H), 3.50 (d, J = 16.4 Hz, 1 H), 3.41 (d, J = 16.4 Hz, 1 H), 2.09–2.00 (m, 2 H), 1.97–1.84 (m, 3 H), 1.83–1.58 (m, 4 H), 1.56–1.46 (m, 1 H), 1.45–1.33 (m, 4 H), 1.31–1.17 (m, 2 H). ^13^C NMR (125.8 MHz, CDCl_3_), ppm: *δ* = 176.76 (s), 138.86 (d), 114.41 (t), 85.22 (s), 49.72 (t), 48.72 (d), 39.34 (t), 33.65 (t), 29.17 (t), 28.39 (t), 28.08 (t), 27.86 (t), 19.79 (t). IR (neat), cm^–1^: 3192 m (br.), 3077 m (br.), 2927 m, 2856 m, 1691 s, 1640 m, 1436 m, 1353 m, 1322 m, 1201 w, 1141 w, 1106 w, 992 m, 908 m, 734 m (br.), 639 m, 616 w. HRMS (Q-Exactive) (*m*/*z*): calcd. for C_13_H_23_N_2_O^+^ [M + H]^+^ 223.18049, found 223.18048.

(5*RS*,6*SR*)-**9b**: ^1^H NMR (500 MHz, CDCl_3_), ppm: *δ* = 7.43 (br. s, 1 H), 5.85–5.74 (m, 1 H), 5.03–4.91 (m, 2 H), 3.49 (m, 2 H), 2.09–1.94 (m, 4 H), 1.91–1.79 (m, 2 H), 1.78–1.64 (m, 3 H), 1.53–1.19 (m, 6 H), 1.18–1.09 (m, 1 H). ^13^C NMR (125.8 MHz, CDCl_3_), ppm: *δ* = 176.73 (s), 138.82 (d), 114.43 (t), 86.18 (s), 49.29 (t), 48.98 (d), 39.59 (t), 33.67 (t), 29.53 (t), 29.43 (t), 29.09 (t), 27.76 (t), 20.51 (t). IR (neat), cm^–1^: 3192 m (br.), 3076 m (br.), 2926 m, 2856 m, 1691 s, 1641 m, 1436 m, 1353 m, 1322 m, 1261 w, 1202 w, 1141 w, 1106 w, 1090 w, 992 m, 908 m, 733 m (br.), 645 m. HRMS (Q-Exactive) (*m*/*z*): calcd. for C_13_H_23_N_2_O^+^ [M + H]^+^ 223.18049, found 223.18048.

#### 3.1.7. Synthesis of (7a′S)-2-(hex-5-en-1-yl)tetrahydrospiro[cyclopentane-1,3′-pyrrolo[1,2-c]imidazol]-1′(2′H)-one (**10a**/**b**)

The reaction was carried out as described for **9** with (±)-**2** (1.73 g, 10 mmol), TEA (2.25 mL, 16 mmol), *l*-prolinamide hydrochloride (2.26 g, 15 mmol) in methanol (125 mL) to give 2.09 g (80%) of the crude product, which represents a typical sample used for headspace analysis.

Column chromatography (SiO_2_, ethyl acetate, then ethyl acetate/ethanol 9:1) afforded several product fractions, which were re-purified (SiO_2_, ethyl acetate) to give 0.22 g of **10a** (containing ca. 10% of the (1*R*,2*R*,7a′*S*)-isomer, which was not further characterised) and 0.48 g of **10b** as slightly yellow, viscous oils. The stereochemistry of the diastereoisomers was assigned in analogy to the chemical shifts recorded in the ^13^C NMR spectra of **7a**–**c** (see earlier).

(1*S*,2*S*,7a′*S*)-**10a**: ^1^H NMR (600 MHz, CDCl_3_), ppm: *δ* = 6.85 (br. s, 1 H), 5.84–5.74 (m, 1 H), 5.03–4.89 (m, 2 H), 3.78 (dd, J = 9.6, 5.8 Hz, 1 H), 2.95–2.88 (m, 1 H), 2.75–2.67 (m, 1 H), 2.18–1.95 (m, 4 H), 1.94–1.86 (m, 1 H), 1.86–1.62 (m, 7 H), 1.62–1.54 (m, 1 H), 1.51–1.43 (m, 1 H), 1.43–1.30 (m, 3 H), 1.24–1.14 (m, 2 H). ^13^C NMR (151.0 MHz, CDCl_3_), ppm: *δ* = 178.94 (s), 139.01 (d), 114.26 (t), 87.00 (s), 65.01 (d), 51.76 (d), 50.35 (t), 33.75 (t), 32.16 (t), 29.45 (t), 29.22 (t), 27.80 (t), 27.59 (t), 27.05 (t), 25.61 (t), 19.84 (t). IR (neat), cm^–1^: 3188 m (br.), 3076 m, 2928 m, 2857 m, 1735 m, 1694 s, 1641 m, 1437 m, 1416 m, 1366 w, 1338 w, 1300 w, 1269 w, 1201 w, 1158 w, 1137 w, 1109 w, 993 m, 908 m, 793 m, 730 w, 697 w. HRMS (Q-Exactive) (*m*/*z*): calcd. for C_16_H_27_N_2_O^+^ [M + H]^+^ 263.21179, found 263.21179.

(1*S*,2*R*,7a′*S*)-**10b**: ^1^H NMR (600 MHz, CDCl_3_), ppm: *δ* = 7.32 (br. s, 1 H), 5.82–5.73 (m, 1 H), 5.01–4.89 (m, 2 H), 3.92 (dd, J = 9.2, 5.8 Hz, 1 H), 3.06–2.99 (m, 1 H), 2.77–2.69 (m, 1 H), 2.18–2.10 (m, 1 H), 2.10–1.99 (m, 3 H), 1.99–1.88 (m, 3 H), 1.86–1.66 (m, 5 H), 1.59–1.50 (m, 1 H), 1.43–1.30 (m, 3 H), 1.30–1.18 (m, 2 H), 1.14–1.05 (m, 1 H). ^13^C NMR (151.0 MHz, CDCl_3_), ppm: *δ* = 179.09 (s), 138.90 (d), 114.32 (t), 87.58 (s), 64.73 (d), 50.83 (d), 50.61 (t), 33.76 (t), 33.22 (t), 29.72 (t), 29.24 (t), 27.71 (t), 27.63 (t), 27.17 (t), 25.54 (t), 20.11 (t). IR (neat), cm^–1^: 3169 m (br.), 3075 m (br.), 2969 m, 2954 w, 2925 m, 2878 m, 2854 m, 2162 w, 1980 w, 1694 s, 1640 m, 1461 w, 1435 m, 1394 w, 1362 m, 1340 w, 1320 w, 1296 w, 1255 w, 1233 w, 1024 w, 1169 w, 1138 m, 1109 w, 1078 w, 1067 w, 1028 w, 994 w, 961 w, 938 w, 908 m, 867 w, 795 m (br.), 704 w, 638 w, 604 w. HRMS (Q-Exactive) (*m*/*z*): calcd. for C_16_H_27_N_2_O^+^ [M + H]^+^ 263.21179, found 263.21167.

#### 3.1.8. Synthesis of 6-hexyl-1,4-diazaspiro[4.4]nonan-2-one (**11a**/**b**)

A mixture of glycinamide hydrochloride (6.77 g, 60 mmol, 2 eq.), TEA (9.3 mL = 9.75 g, 66 mmol) and 2-hexylcyclopentan-1-one ((±)-**3**, 5.31 g, 30 mmol) in methanol (50 mL) was heated under reflux for 24 h. After cooling to room temperature, a white precipitate was formed. The reaction mixture was filtered, and the solvent was evaporated under reduced pressure. Water (50 mL) was added and the mixture extracted with ethyl acetate (2 × 100 mL). The combined organic phases were washed with water (50 mL) and a saturated aqueous solution of NaCl (50 mL), dried (Na_2_SO_4_), filtered and concentrated under reduced pressure. Column chromatography (SiO_2_, *n*-heptane/ethyl acetate 7:3, then ethyl acetate and ethyl acetate/ethanol 1:1) afforded 3.48 g (52%) of a mixture of diastereoisomers (ca. 1:1), which represents a typical sample used for headspace analysis.

(5*RS*,6*RS*)-**11a** and (5*RS*,6*SR*)-**11b**: ^1^H NMR (600 MHz, CDCl_3_), ppm: *δ* = 7.76 and 7.28 (br. s, 1 H), 3.51 and 3.49 (d, J = 16.6 and 16.2, 1 H), 3.47 and 3.41 (d, J = 16.6 and 16.2, 1 H), 2.05–1.60 (m, 7 H), 1.54–1.08 (m, 11 H), 0.88 (t, J = 6.7, 3 H). ^13^C NMR (150.9 MHz, CDCl_3_), ppm: *δ* = 176.99 and 176.87 (s), 86.32 and 85.33 (s), 49.83 and 49.37 (t), 49.03 and 48.68 (d), 39.62 and 39.32 (t), 31.83 and 31.80 (t), 29.67 (t), 29.58 and 29.42 (t), 28.41 and 28.39 (t), 28.31 and 28.27 (t), 22.64 and 22.63 (t), 20.53 and 19.81 (t), 14.09 (q). HRMS (Q-Exactive) (*m*/*z*): calcd. for C_13_H_25_N_2_O^+^ [M + H]^+^ 225.19614, found 225.19601 and 225.19618.

### 3.2. Isomerisations in Methanol

Profragrances **4a**/**b** and **7a**–**c** (ca. 30 mg) were dissolved in methanol-d_4_ (0.7 mL) with trimethylsilane as the internal standard. Quantitative ^1^H NMR spectra (d1 = 20 s) were recorded over 35 (**4**) or 55 days (**7**) with more than 15 data points for each sample. In the measurement of (1*R*,2*R*,7a′*S*)-**7**, some of the data were not measured but extrapolated because the *l*-prolinamide peak overlapped with the peak of **7c** and thus could not be integrated in the first spectra of the series.

### 3.3. Dynamic Headspace Measurements on Dry Cotton Sheets

#### 3.3.1. Fabric Softening Procedure

To an unperfumed fabric softening formulation consisting of a quaternised triethanolamine esterquat (Stepantex^®^ VL90A, 16.5 wt-%), an aqueous solution (10%) of calcium chloride (0.6 wt-%) and water (82.9 wt-%) were added imidazolidin-4-one-derived profragrances **4**–**11** (at 0.014 mmol g^−1^ of softener), roughly corresponding to a total amount of 0.1 wt-% of fragrance to be released with respect to the quantity of softener. Typically, the profragrances (0.1 mmol) were dissolved in ethyl acetate or ethanol (0.2 mL) and completed with the softener formulation to 7 g. The mixture was vigorously shaken (10 times). The formulation with the profragrances (70 mg) was then pipetted into a small bottle (250 mL) and diluted with water (23 g). The bottle was shaken (10 times), and then a cotton sheet (Eidgenössische Materialprüfanstalt (EMPA) test cloth Nr. 221) was added that had been pre-washed with an unperfumed detergent powder and cut to ca. 15 × 15 cm squares, each weighing ca. 5.1 g). The bottle was closed, shaken manually for 3 min and then left standing for 2 min. The cotton sheet was taken out, wrung out by hand (to be as close as possible to 10.0 g) and line dried in a closed cupboard for 1 day or for 3 days (at an ambient humidity of 60–65% at 22°C). Reference samples containing an equimolar amount of cyclopentanone derivatives (±)-**1**–(±)-**3** to be released from the corresponding profragrances were prepared and analysed in the same way [35,39,49].

#### 3.3.2. Dynamic Headspace Sampling

The line-dried cotton sheets were placed in a homemade sampling cell (ca. 165 mL inner volume), which was thermostatted at 25 °C. A continuous flow of air (ca. 200 mL min^−1^) was aspirated through activated charcoal (to avoid contamination of the air) and through a saturated aqueous solution of NaCl (to ensure a constant humidity of 75%) and then through the sampling cell with the cotton sheet. The volatiles evaporating from the sample were adsorbed onto waste and clean cartridges, each containing 100 mg of poly(2,6-diphenyl-*p*-phenylene oxide) (Tenax^®^ TA). After sampling onto a waste cartridge for 15 min (equilibration) and onto a clean cartridge for 15 min (data point 1), the volatiles were alternately trapped onto a waste cartridge for 45 min and onto a clean cartridge for 15 min (7 times, data points 2–8). Waste cartridges were discarded; clean cartridges were thermally desorbed and analysed by GC. All measurements were carried out at least in duplicate; slight variations with respect to previously reported data [39] are due to additional measurements.

## 4. Conclusions

We demonstrated that imidazolidin-4-one derivatives are suitable profragrances for the controlled release of volatile cyclopentanone-based fragrances in functional perfumery applications. Depending on the amino acid amide chosen as the starting material, bicyclic 1,4-diazaspiro[4.4]nonan-2-ones (**4**–**6**, **9** and **11**) or tricyclic tetrahydrospiro[cyclopentane-1,3′-pyrrolo[1,2-*c*]imidazol]-1′(2′*H*)-ones (**7** and **10**) and tetrahydro-2′*H*-spiro[cyclopentane-1,3′-imidazo[1,5-*a*]pyridin]-1′(5′*H*)-ones (**8**) were obtained as mixtures of diastereoisomers. Column chromatography allowed at least partial separation of the stereoisomers. Their structures were assigned by NMR spectroscopy and, in some cases, supported by DFT calculations of their relative energies. The diastereoisomers isomerised in polar solution under thermodynamic control to form an equilibrium with a given diastereoisomeric distribution. Dynamic headspace analysis on dry cotton showed that the profragrances efficiently released the cyclopentanone fragrances. In particular, tricyclic tetrahydrospiro[cyclopentane-1,3′-pyrrolo[1,2-*c*]imidazol]-1′(2′*H*)-ones **7** and **10** increased the headspace concentration of the released fragrance above the cotton surface by up to 100 times with respect to the unmodified reference compound.

In view of an increasing demand from consumers for eco-friendly delivery systems, bio-sourced amino acid derivatives are of particular interest as substrates for the preparation of biocompatible profragrances. The present work represents an important milestone in this direction.

## 5. Patents

Parts of the present work are the subject of a patent application (WO 2021/209396; cited as ref. [39] in this manuscript).

## Figures and Tables

**Table 1:** Calculated relative energies and Boltzmann distributions for diastereoisomers **7a**–**d** and **8a**–**d** in comparison to the isolated ratio of isomers.

Compound	Stereochemistry	Relative Energy [kJ]	Boltzmann Calc. [%]	Experimental Ratio [%]	Recorded ^13^C NMR Shift [ppm]
**7a**	(1*S*,2*S*,7a′*S*)	1.90	31	36	87.1
**7b**	(1*S*,2*R*,7a′*S*)	0.00	66	52	87.7
**7c**	(1*R*,2*R*,7a′*S*)	7.78	3	10	86.7
**7d**	(1*R*,2*S*,7a′*S*)	10.26	1	2	88.6
(±)-**8a**	(1*RS*,2*RS*,8a′*SR*)	0.00	91	71	86.0
(±)-**8b**	(1*SR*,2*SR*,8a′*SR*)	7.74	4	10	85.0
(±)-**8c**	(1*RS*,2*SR*,8a′*SR*)	6.98	5	19	86.1
(±)-**8d**	(1*SR*,2*RS*,8a′*SR*)	22.91	0.01	n.d.	n.d.

## Data Availability

All available data are contained in the present manuscript and the Supplementary Materials of this article.

## Supplementary Materials

The following supporting information can be downloaded at: https://www.mdpi.com/article/10.3390/molecules28010382/s1. Description of instrumentation and methods used; Figures S1–S19: ^1^H and ^13^C NMR spectra of compounds **4**–**11**; Tables S1–S5: numerical data for Scheme 6 and Scheme 7; Tables S6–S9: numerical data for Figure 6 and Figure 7.

## Sample Availability

Samples of the compounds are not available from the authors.

## References

[1] Herz R.S., Larsson M., Trujillo R., Casola M.C., Ahmed F.K., Lipe S., Brashear M.E. (2022). A three-factor benefits framework for understanding consumer preference for scented household products: Psychological interactions and implications for future development. Cognit. Res. Princ. Implic..

[2] Milotic D. (2003). The impact of fragrance on consumer choice. J. Consum. Behav..

[3] He L., Hu J., Deng W. (2018). Preparation and application of flavor and fragrance capsules. Polym. Chem..

[4] Kaur R., Kukkar D., Bhardwaj S.K., Kim K.-H., Deep A. (2018). Potential use of polymers and their complexes as media for storage and delivery of fragrances. J. Control. Release.

[5] Bruyninckx K., Dusselier M. (2019). Sustainable chemistry considerations for the encapsulation of volatile compounds in laundry-type applications. ACS Sustain. Chem. Eng..

[6] Perinelli D.R., Palmieri G.F., Cespi M., Bonacucina G. (2020). Encapsulation of flavours and fragrances into polymeric capsules and cyclodextrins inclusion complexes: An update. Molecules.

[7] Wei M., Pan X., Rong L., Dong A., He Y., Song X., Li J. (2020). Polymer carriers for controlled fragrance release. Mater. Res. Express.

[8] Tian Q., Zhou W., Cai Q., Ma G., Lian G. (2021). Concepts, processing, and recent developments in encapsulating essential oils. Chin. J. Chem. Eng..

[9] Mamusa M., Resta C., Sofroniou C., Baglioni P. (2021). Encapsulation of volatile compounds in liquid media: Fragrances, flavors, and essential oils in commercial formulations. Adv. Colloid Interface Sci..

[10] Xiao Z., Sun P., Liu H., Zhao Q., Niu Y., Zhao D. (2022). Stimulus responsive microcapsules and their aromatic applications. J. Control. Release.

[11] Zaitoon A., Luo X., Lim L.-T. (2022). Triggered and controlled release of active gaseous/volatile compounds for active packaging applications of agri-food products: A review. Compr. Rev. Food Sci. Food Saf..

[12] Herrmann A. (2007). Controlled release of volatiles under mild reaction conditions: From nature to everyday products. Angew. Chem. Int. Ed..

[13] Herrmann A., Herrmann A. (2010). Profragrances and properfumes. The Chemistry and Biology of Volatiles.

[14] Herrmann A. (2017). Profragrance chemistry as an interdisciplinary research area and key technology for fragrance delivery. Chimia.

[15] Herrmann A. (2019). Controlled release of volatile compounds using the Norrish type II reaction. Photochemistry.

[16] Liang Z., Fang Z., Pai A., Luo J., Gan R., Gao Y., Lu J., Zhang P. (2022). Glycosidically bound aroma precursors in fruits: A comprehensive review. Crit. Rev. Food Sci. Nutr..

[17] Blackmore T.R., Thompson P.E. (2011). Imidazolidin-4-ones: Their syntheses and applications. Heterocycles.

[18] Swain S.P., Mohanty S. (2019). Imidazolidinones and imidazolidine-2,4-diones as antiviral agents. ChemMedChem.

[19] Klixbüll U., Bundgaard H. (1984). Prodrugs as drug delivery systems. XXX. 4-Imidazolidinones as potential bioreversible derivatives for the α-aminoamide moiety in peptides. Int. J. Pharm..

[20] Bak A., Fich M., Larsen B.D., Frokjaer S., Friis G.J. (1999). *N*-Terminal 4-imidazolidinone prodrugs of Leu-enkephalin: Synthesis, chemical and enzymatic stability studies. Eur. J. Pharm. Sci..

[21] Rasmussen G.J., Bundgaard H. (1991). Prodrugs of peptides. 15. 4-Imidazolidinone prodrug derivatives of enkephalins to prevent aminopeptidase-catalyzed metabolism in plasma and absorptive mucosae. Int. J. Pharm..

[22] Rasmussen G.J., Bundgaard H. (1991). Prodrugs of peptides. 10. Protection of di- and tripeptides against aminopeptidase by formation of bioreversible 4-imidazolidinone derivatives. Int. J. Pharm..

[23] Chambel P., Capela R., Lopes F., Iley J., Morais J., Gouveia L., Gomes J.R.B., Gomes P., Moreira R. (2006). Reactivity of imidazolidin-4-one derivatives of primaquine: Implications for prodrug design. Tetrahedron.

[24] Vale N., Nogueira F., do Rosário V.E., Gomes P., Moreira R. (2009). Primaquine dipeptide derivatives bearing an imidazolidin-4-one moiety at the *N*-terminus as potential antimalarial prodrugs. Eur. J. Med. Chem..

[25] Weng Larsen S., Sidenius M., Ankersen M., Larsen C. (2003). Kinetics of degradation of 4-imidazolidinone prodrug types obtained from reacting prilocaine with formaldehyde and acetaldehyde. Eur. J. Pharm. Sci..

[26] Durbin A.K., Rydon H.N. (1970). The equilibrium between the antibiotics hetacillin and ampicillin in solution. J. Chem. Soc. D Chem. Commun..

[27] Tsuji A., Yamana T. (1974). Kinetic approach to the development in β-lactam antibiotics. II. Prodrug. (1). Simultaneous determination of hetacillin and ampicillin, and its application to the stability of hetacillin in aqueous solution. Chem. Pharm. Bull..

[28] Klixbüll U., Bundgaard H. (1985). Kinetics of reversible reactions of ampicillin with various aldehydes and ketones with formation of 4-imidazolidinones. Int. J. Pharm..

[29] Gomes P., Araújo M.J., Rodrigues M., Vale N., Azevedo Z., Iley J., Chambel P., Morais J., Moreira R. (2004). Synthesis of imidazolidin-4-one and 1*H*-imidazo[2,1-*a*]isoindole-2,5(3*H*,9b*H*)-dione derivatives of primaquine: Scope and limitations. Tetrahedron.

[30] Giorgioni G., Claudi F., Ruggieri S., Ricciutelli M., Palmieri G.F., Di Stefano A., Sozio P., Cerasa L.S., Chiavaroli A., Ferrante C. (2010). Design, synthesis, and preliminary pharmacological evaluation of new imidazolinones as L-DOPA prodrugs. Bioorg. Med. Chem..

[31] Friedli F.E., Koehle H.J., Fender M., Watts M., Keys R., Frank P., Toney C.J., Doerr M. (2002). Upgrading triethanolamine esterquat performance to new levels. J. Surfactants Deterg..

[32] Mishra S., Tyagi V.K. (2007). Ester quats: The novel class of cationic fabric softeners. J. Oleo Sci..

[33] Escher S.D., Oliveros E. (1994). A quantitative study of factors that influence the substantivity of fragrance chemicals on laundered and dried fabrics. J. Am. Oil Chem. Soc..

[34] Obendorf S.K., Liu H., Tan K., Leonard M.J., Young T.J., Incorvia M.J. (2009). Adsorption of aroma chemicals on cotton fabric in different aqueous environments. J. Surfactants Deterg..

[35] Trachsel A., Buchs B., Godin G., Crochet A., Fromm K.M., Herrmann A. (2012). Preparation of imidazolidin-4-ones and their evaluation as hydrolytically cleavable precursors for the slow release of bioactive volatile carbonyl derivatives. Eur. J. Org. Chem..

[36] Surburg H., Panten J. (2016). Common Fragrance and Flavor Materials—Preparation, Properties and Uses.

[37] Coulomb J., Demole E. (2020). (to Firmenich SA). Cyclopentanone compounds. PCT Int. Patent Appl..

[38] Arctander S. (1969). Perfume and Flavor Chemicals.

[39] Herrmann A., Lamboley S. (2021). (to Firmenich SA). Compounds for providing a long-lasting odor. PCT Int. Patent Appl..

[40] Koch W., Holthausen M.C. (2002). A Chemist’s Guide to Density Functional Theory.

[41] Sholl D.S., Steckel J.A. (2009). Density Functional Theory—A Practical Introduction.

[42] (2021). Schrödinger Release 2022-3: MacroModel.

[43] (2018). Spartan’18.

[44] Godin G., Levrand B., Trachsel A., Lehn J.-M., Herrmann A. (2010). Reversible formation of aminals: A new strategy to control the release of bioactive volatiles from dynamic mixtures. Chem. Commun..

[45] Buchs B., Godin G., Trachsel A., de Saint-Laumer J.-Y., Lehn J.-M., Herrmann A. (2011). Reversible aminal formation: Controlling the evaporation of bioactive volatiles by dynamic combinatorial/covalent chemistry. Eur. J. Org. Chem..

[46] Herrmann A. (2012). Dynamic mixtures: Challenges and opportunities for the amplification and sensing of scents. Chem. Eur. J..

[47] Rouseff R.L., Cadwallader K.R. (2001). Headspace Analysis of Food and Flavors: Theory and Practice.

[48] Rubiolo P., Sgorbini B., Liberto E., Cordero C., Bicchi C., Herrmann A. (2010). Analysis of the plant volatile fraction. The Chemistry and Biology of Volatiles.

[49] Lamboley S., Trachsel A., Herrmann A. (2020). Polystyrene-based 2-oxoacetates for the light-induced release of fragrances under realistic application conditions. Macromol. Chem. Phys..

