# Supplementary material for: Release of Volatile Cyclopentanone Derivatives from Imidazolidin-4-One Profragrances in a Fabric Softener Application
Source: Molecules. 2023 Jan 2;28(1):382. doi: 10.3390/molecules28010382 (PMC9822342; doi:10.3390/molecules28010382)
Supplement: Supplementary file 1 [file molecules-28-00382-s001.zip › molecules-2133826-supplementary.pdf]

molecules

MDPI

Supplementary Material

Release of Volatile Cyclopentanone Derivatives from Imidazolidin-4-One Profragrances in a Fabric Softener Application

Serge Lamboley, Basile Vuichoud, Jean-Yves de Saint Laumer and Andreas Herrmann *

Firmenich SA, Division Recherche et Développement, Rue de la Bergère 7, 1242 Satigny, Switzerland
* Correspondence: andreas.herrmann@firmenich.com

Instrumentation and Methods

Nuclear magnetic resonance (NMR) spectra were measured on Bruker Avance III 500 and 600 spectrometers; the chemical displacements δ are indicated in ppm with respect to $Si(CH_3)_4$ (TMS) as the internal standard. Quantitative ^1H NMR spectra (d1 = 20 s) were recorded on an instrument (500 MHz) equipped with a BB/H&F cryoprobe and an AVIII console. Multiplets indicated together with the chemical shifts obtained from decoupled ^{13}C NMR spectra were determined by distortionless enhancement of polarisation transfer (DEPT) measurements; two sets of spectra (DEPT-90 and DEPT-135, data not shown) were systematically recorded. Nuclear Overhauser Effect Spectroscopy (NOESY) experiments and density functional theory (DFT) calculations using SPARTAN were carried out to determine the stereochemistry of the molecules.

Infrared (IR) spectra were measured on a Perkin Elmer Frontier FTIR spectrometer, with maxima given in cm^{-1} and the intensities of the IR bands indicated as strong (s), medium (m) or weak (w), sh = shoulder.

High-resolution mass spectra (HRMS) were recorded on a Thermo Fisher Scientific Q-Exactive system (0726020) equipped with a heated electrospray ionisation (H-ESI) probe source operated in the full scan/MS2 mode (m/z from 100 to 1500) with the H-ESI probe source in the positive or negative mode, depending on the ionisation potential of the molecule, and at a spray voltage of 3500 V with a resolution of 35000 (full scan) and 17500 (MS2). A Waters Acquity I-Class Ultra Performance Liquid Chromatography (UPLC) system equipped with an Acquity UPLC BEH C18 1.7 mm column with dimensions of 2.1 × 100 mm was coupled to the Thermo Fisher Q-Exactive system using a 9 min elution program at a flow rate of 0.4 mL min^{-1} with water (containing 0.1% formic acid) as solvent A and acetonitrile (containing 0.1% formic acid) as solvent B, starting at 98% A from 0 to 1 min, 98% A to 100% B from 1 to 6 min, 100% B from 6 to 7 min, 100% B to 98% A from 7 to 8 min, and 98% A from 8 to 9 min. Alternatively, direct solvent injections were performed at a flow rate of 0.4 mL min^{-1} with acetonitrile (containing 0.1% of formic acid) for 5 min, using the same Q-Exactive system in full scan mode at a resolution of 70000.

Dynamic headspace measurements were carried out on a Perkin Elmer TurboMatrix ATD thermodesorber coupled to an Agilent® Technologies 7890A GC System with a flame ionisation detector (FID). The volatiles were eluted with He on an Agilent® DB-1 capillary column (30 m × 0.32 µm, film 0.25 µm) by using a temperature gradient from 60°C to 200°C at 15°C min^{-1}. Headspace concentrations (in ng L^{-1}) were obtained by external standard calibration by injecting solutions of known amounts of cyclopentanone derivatives (±)-**1**–(±)-**3** onto clean Tenax® cartridges and desorbing them as described earlier.

Figures

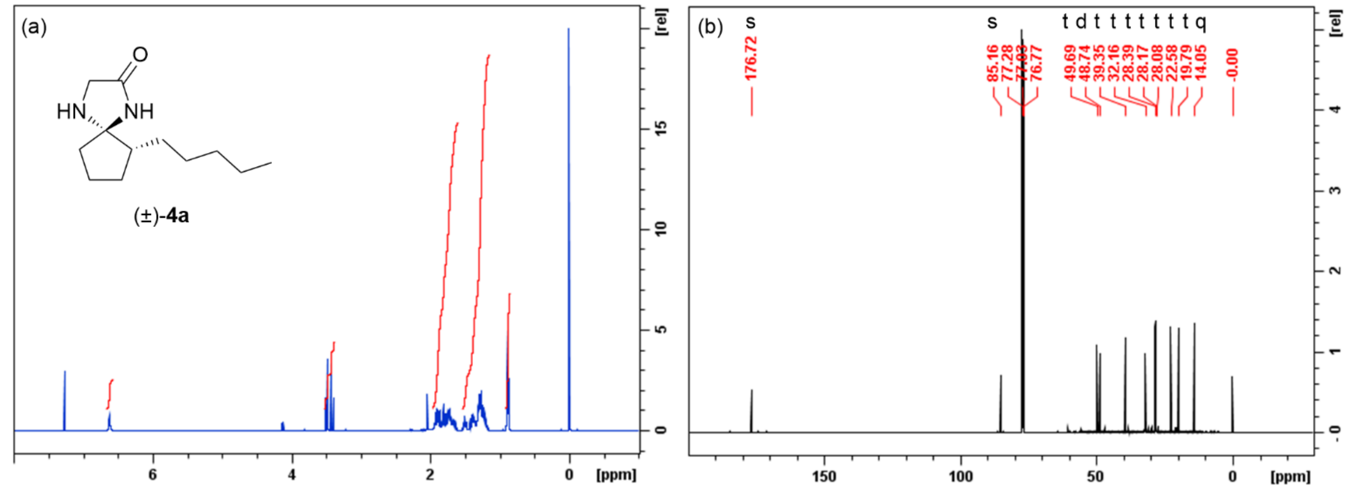

Figure S1. (a) ^{1}H NMR spectrum (500 MHz, $CDCl_3$) and (b) ^{13}C NMR spectrum (125.8 MHz, $CDCl_3$) of (5*RS*,6*RS*)-**4a**. The compound contains ca. 2% of (5*RS*,6*SR*)-**4b**.

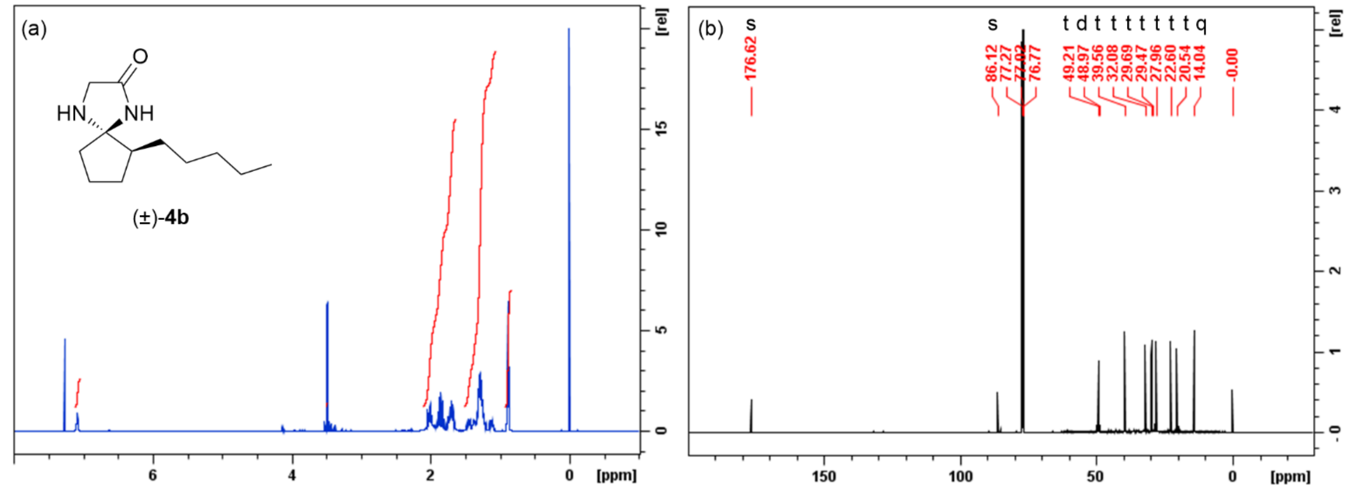

Figure S2. (a) ^{1}H NMR spectrum (500 MHz, $CDCl_3$) and (b) ^{13}C NMR spectrum (125.8 MHz, $CDCl_3$) of (5*RS*,6*SR*)-**4b**. The compound contains ca. 7% of (5*RS*,6*RS*)-**4a**.

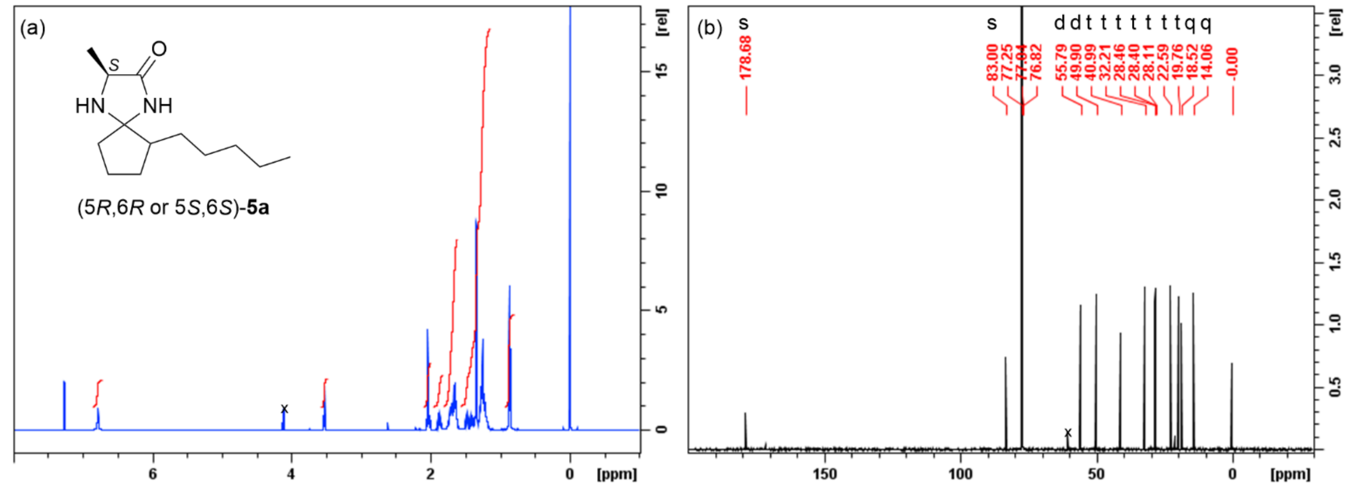

Figure S3. (a) ^{1}H NMR spectrum (600 MHz, $CDCl_3$) and (b) ^{13}C NMR spectrum (150.9 MHz, $CDCl_3$) of (3*S*,5*R*,6*R* or 3*S*,5*S*,6*S*)-**5a**. The compound contains small amounts of ethyl acetate (labelled as x).

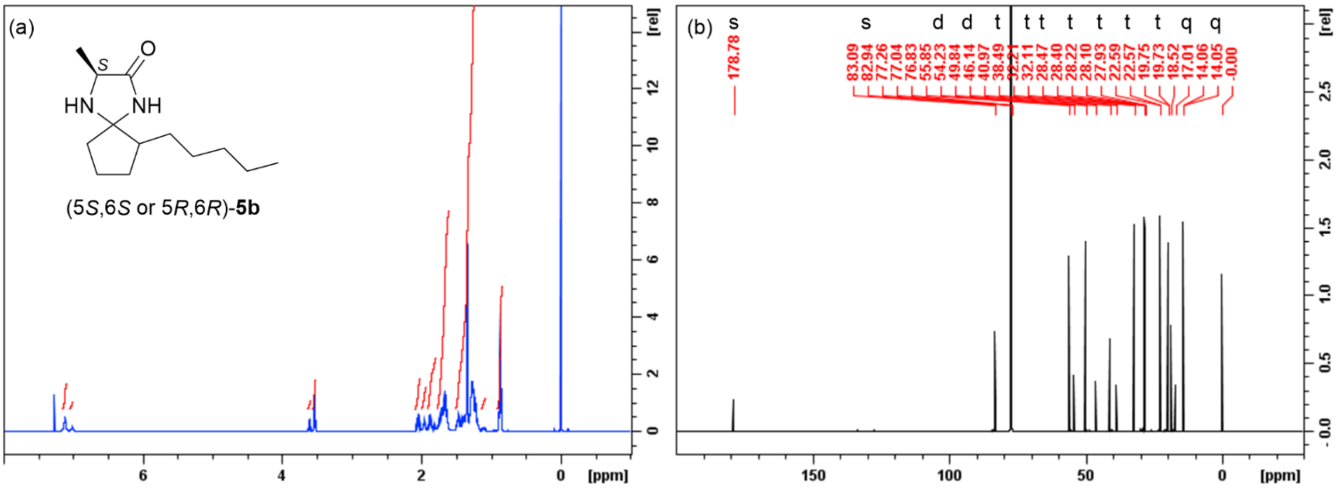

Figure S4. (a) ^{1}H NMR spectrum (600 MHz, $CDCl_3$) and (b) ^{13}C NMR spectrum (150.9 MHz, $CDCl_3$) of (3*S*,5*S*,6*S* or 3*S*,5*R*,6*R*)-**5b**, minor isomer (ca. 26%) in a mixture with **5a** (ca. 74%).

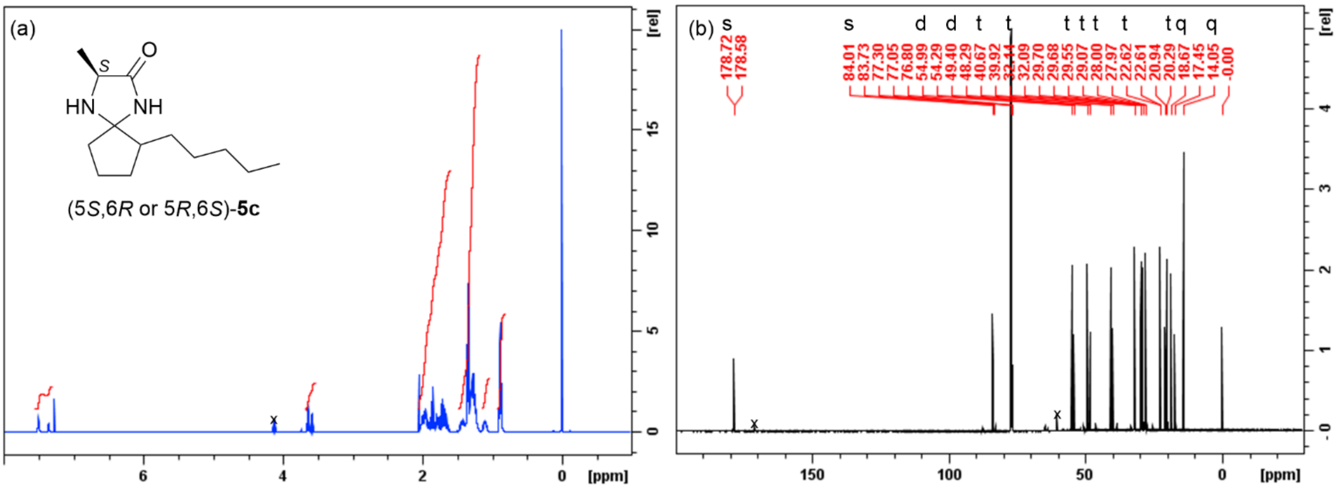

Figure S5. (a) ^{1}H NMR spectrum (500 MHz, $CDCl_3$) and (b) ^{13}C NMR spectrum (125.8 MHz, $CDCl_3$) of (3*S*,5*S*,6*R* or 3*S*,5*R*,6*S*)-**5c**, major isomer (ca. 64%) in a mixture with **5d** (ca. 36%). The compound contains small amounts of ethyl acetate (labelled as x).

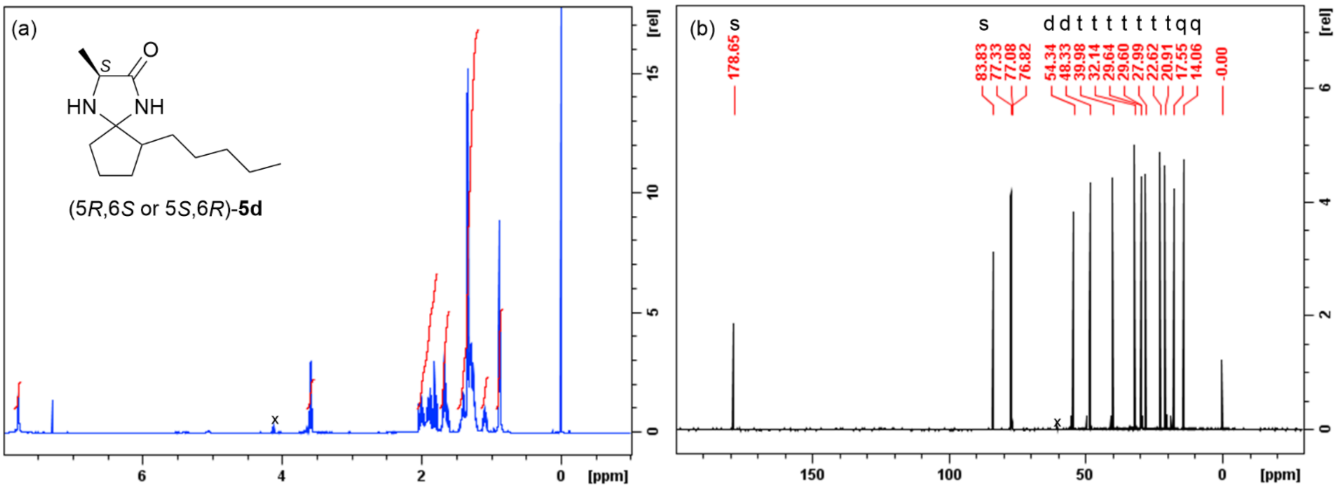

Figure S6. (a) ^{1}H NMR spectrum (500 MHz, $CDCl_3$) and (b) ^{13}C NMR spectrum (125.8 MHz, $CDCl_3$) of (3*S*,5*R*,6*S* or 3*S*,5*S*,6*R*)-**5d**. The compound contains small amounts of ethyl acetate (labelled as x).

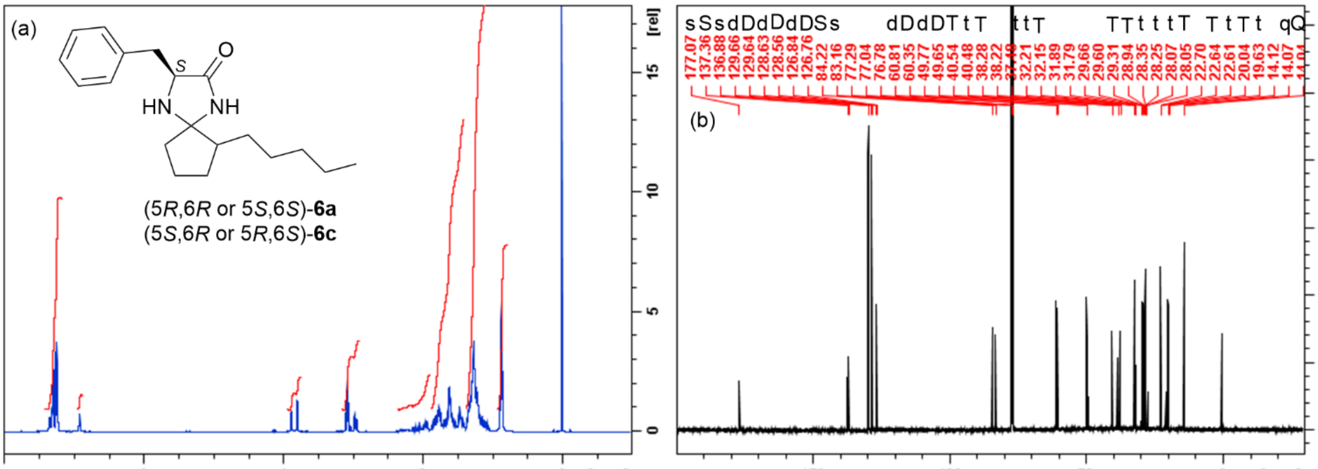

Figure S7. (a) ^{1}H NMR spectrum (500 MHz, $CDCl_3$) and (b) ^{13}C NMR spectrum (125.8 MHz, $CDCl_3$) of (3*S*,5*R*,6*R* or 3*S*,5*S*,6*S*)-**6a** and (3*S*,5*S*,6*R* or 3*S*,5*R*,6*S*)-**6c** (ca. 50:50). The compound contains small amounts of 2-pentylcyclopentan-1-one.

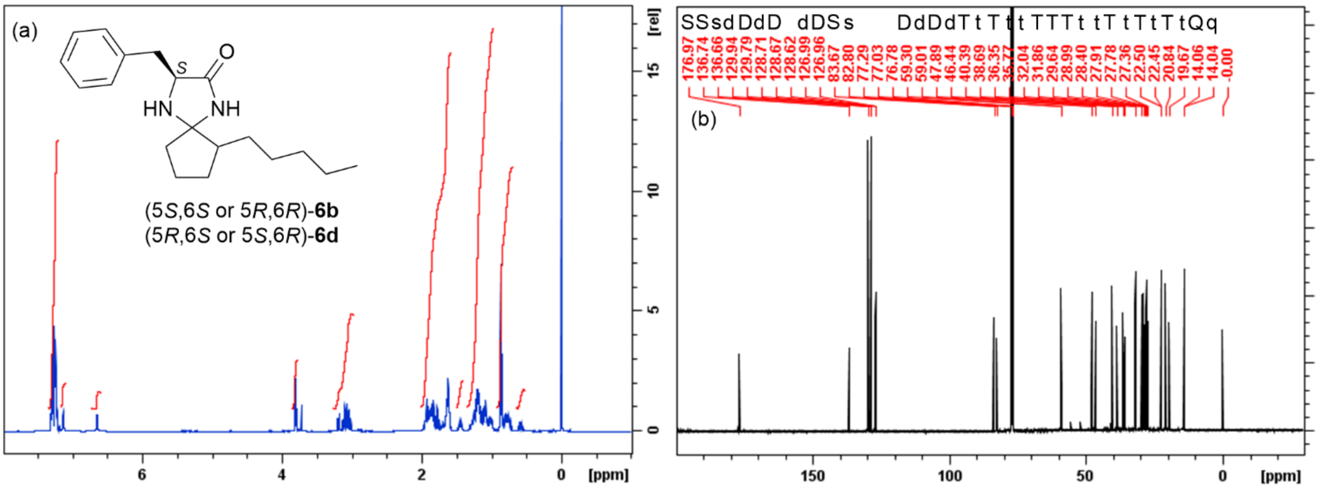

Figure S8. (a) ^{1}H NMR spectrum (500 MHz, $CDCl_3$) and (b) ^{13}C NMR spectrum (125.8 MHz, $CDCl_3$) of (3*S*,5*S*,6*S* or 3*S*,5*R*,6*R*)-**6b** and (3*S*,5*R*,6*S* or 3*S*,5*S*,6*R*)-**6d** (ca. 45:55).

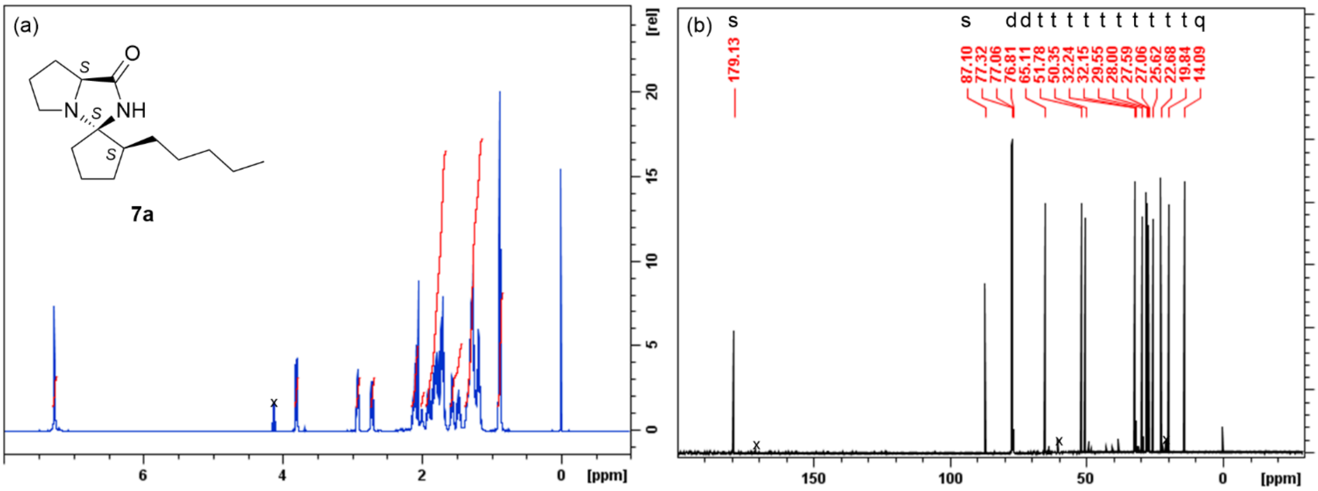

Figure S9. (a) ^{1}H NMR spectrum (500 MHz, $CDCl_3$) and (b) ^{13}C NMR spectrum (125.8 MHz, $CDCl_3$) of (1*S*,2*S*,7a′*S*)-**7a**. The compound contains small amounts of ethyl acetate (labelled as x).

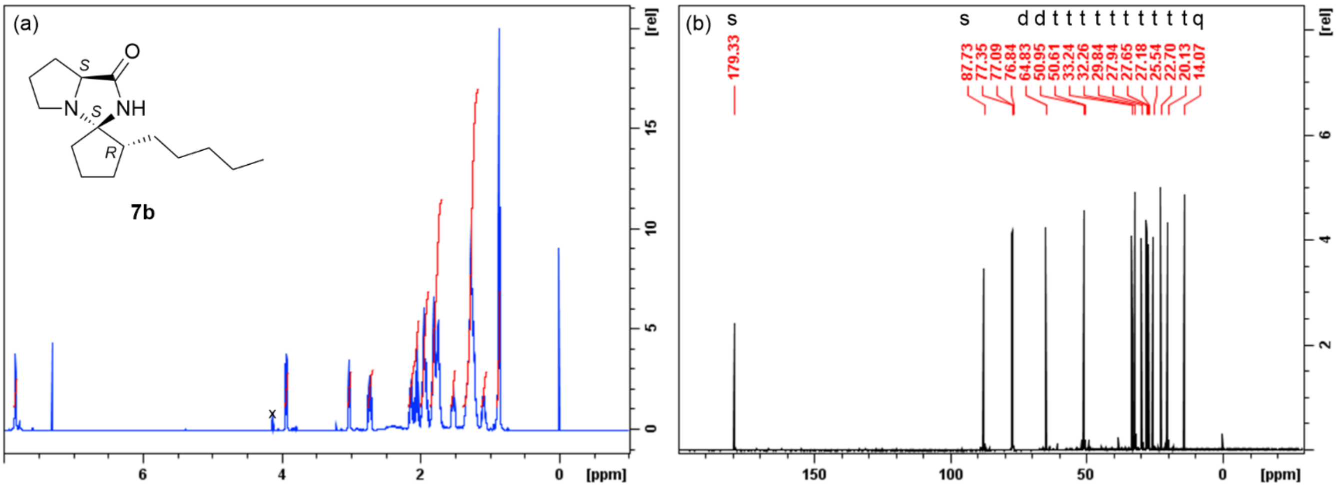

Figure S10. (a) ^1H NMR spectrum (500 MHz, $CDCl_3$) and (b) ^{13}C NMR spectrum (125.8 MHz, $CDCl_3$) of (1*S*,2*R*,7a′*S*)-**7b**. The compound contains ca. 3% of (1*S*,2*R*,7a′*S*)-**7a** and small amounts of ethyl acetate (labelled as x).

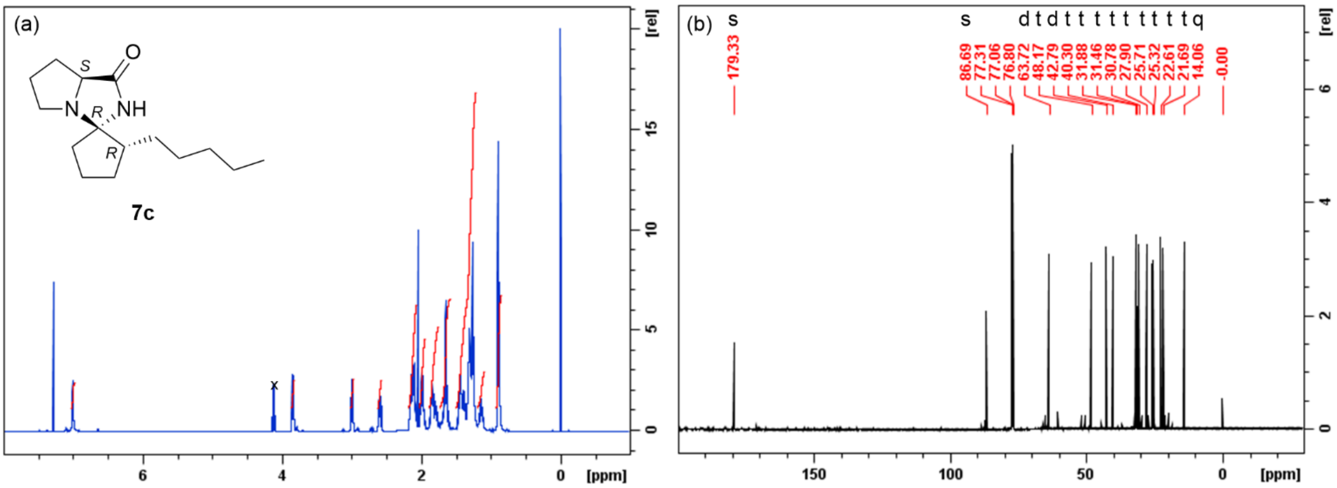

Figure S11. (a) ^1H NMR spectrum (500 MHz, $CDCl_3$) and (b) ^{13}C NMR spectrum (125.8 MHz, $CDCl_3$) of (1*R*,2*R*,7a′*S*)-**7c**. The compound contains ca. 7% of (1*S*,2*R*,7a′*S*)-**7a** and small amounts of ethyl acetate (labelled as x).

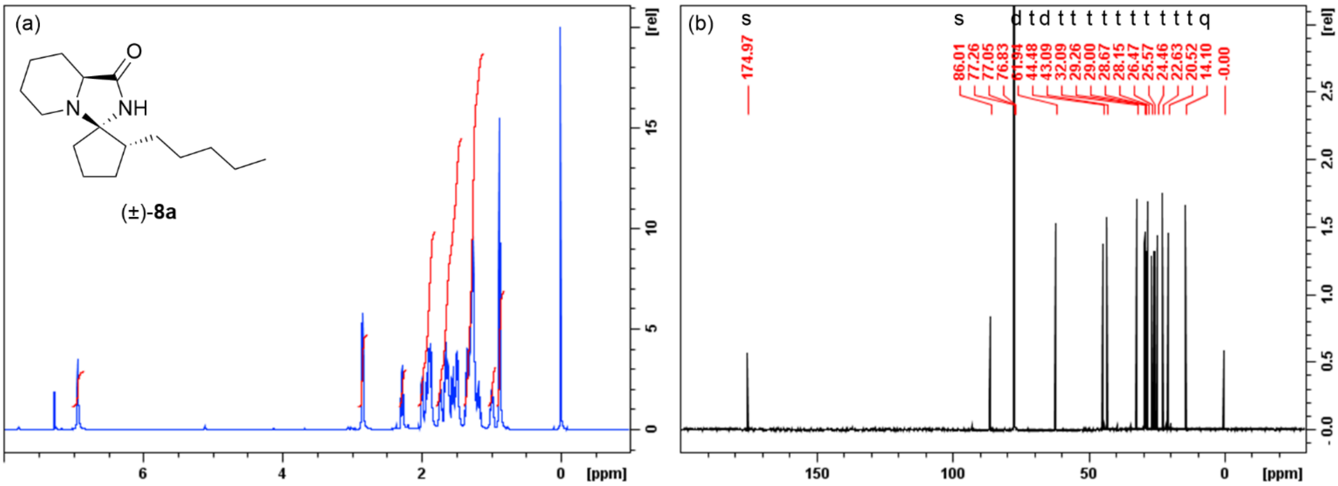

Figure S12. (a) ^1H NMR spectrum (600 MHz, $CDCl_3$) and (b) ^{13}C NMR spectrum (151.0 MHz, $CDCl_3$) of (1*RS*,2*RS*,8a'*SR*)-**8a**.

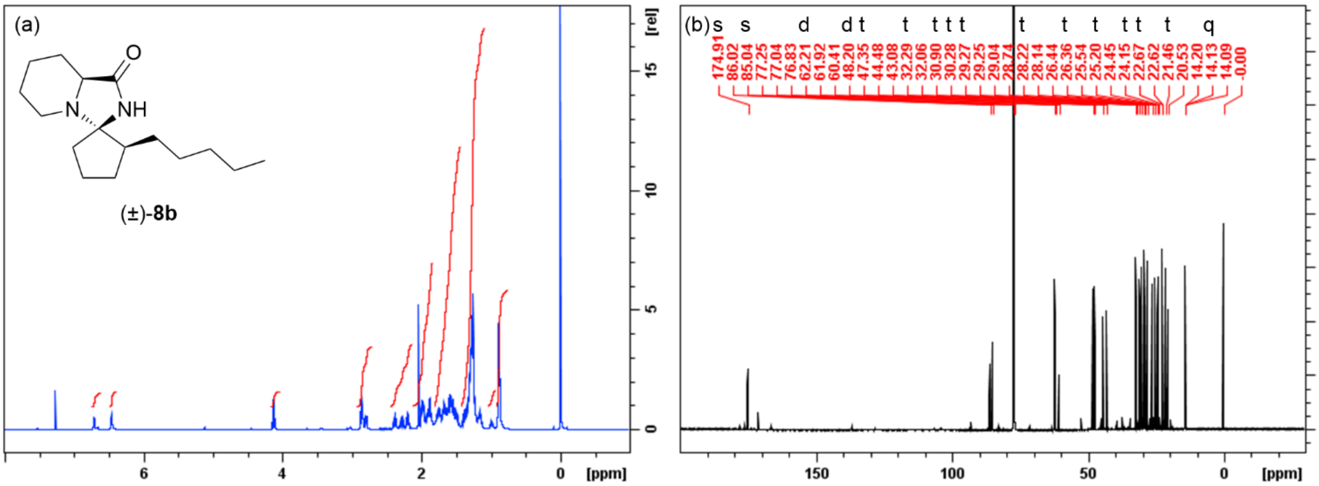

Figure S13. (a) ^{1}H NMR spectrum (600 MHz, $CDCl_3$) and (b) ^{13}C NMR spectrum (151.0 MHz, $CDCl_3$) of (1*SR*,2*SR*,8a'*SR*)-**8b**, major isomer (ca. 56%) in a mixture with (1*RS*,2*RS*,8a'*SR*)-**8a** (ca. 44%), and some impurities.

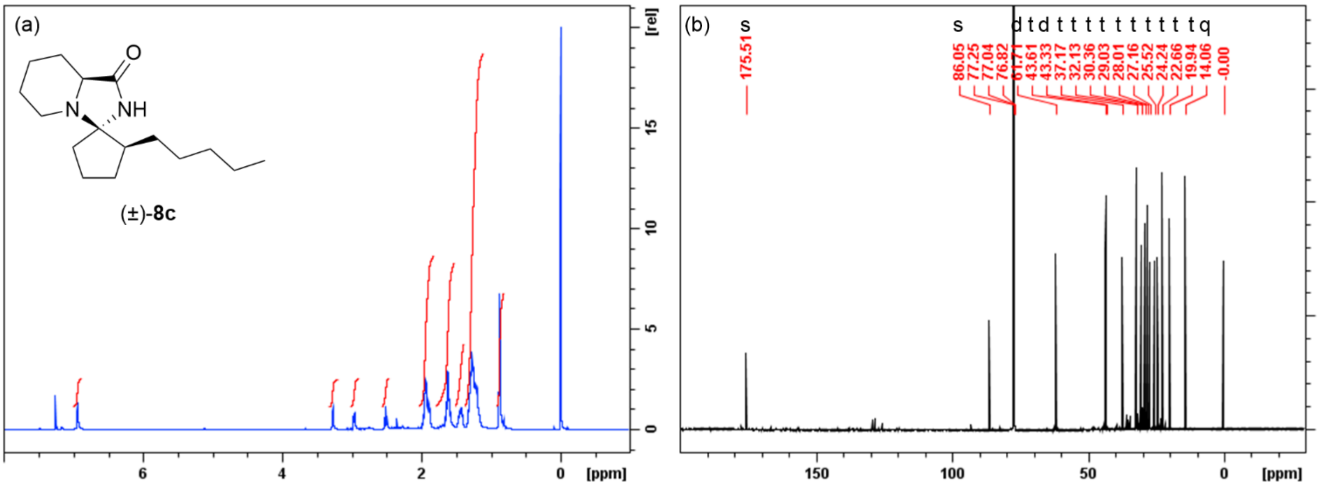

Figure S14. (a) ^{1}H NMR spectrum (600 MHz, $CDCl_3$) and (b) ^{13}C NMR spectrum (151.0 MHz, $CDCl_3$) of (1*RS*,2*SR*,8a'*SR*)-**8c**.

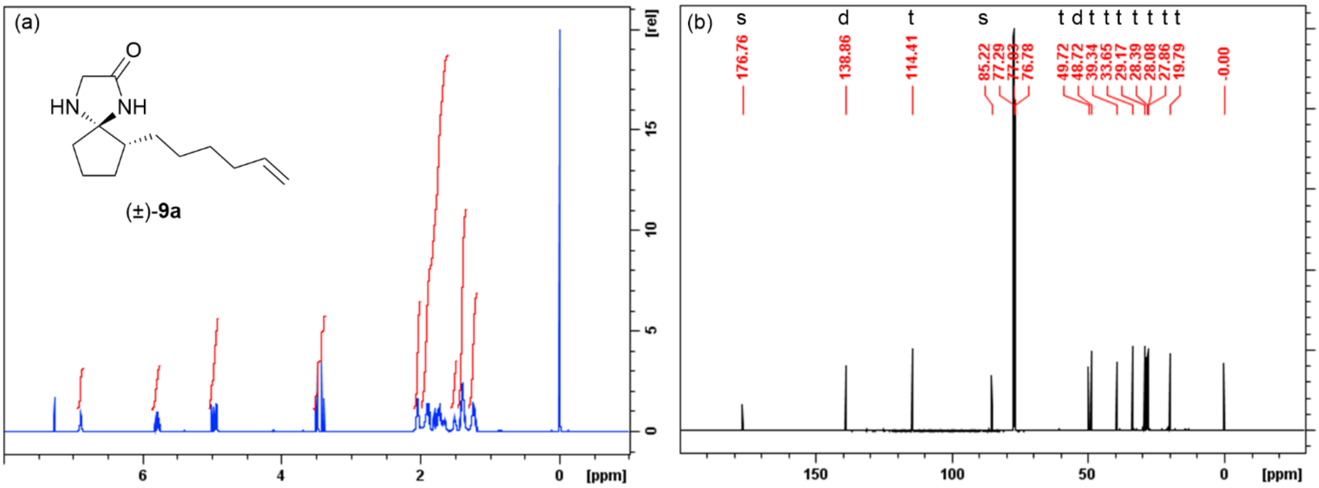

Figure S15. (a) ^{1}H NMR spectrum (500 MHz, $CDCl_3$) and (b) ^{13}C NMR spectrum (125.8 MHz, $CDCl_3$) of (5*RS*,6*RS*)-**9a**.

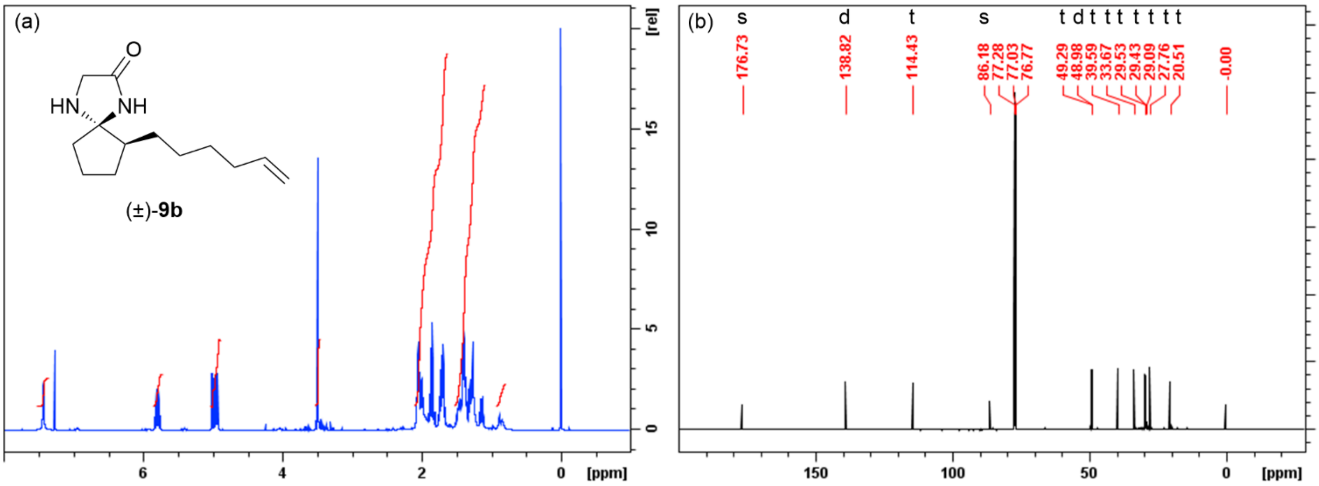

Figure S16. (a) ^{1}H NMR spectrum (500 MHz, $CDCl_3$) and (b) ^{13}C NMR spectrum (125.8 MHz, $CDCl_3$) of (5*RS*,6*SR*)-**9b**.

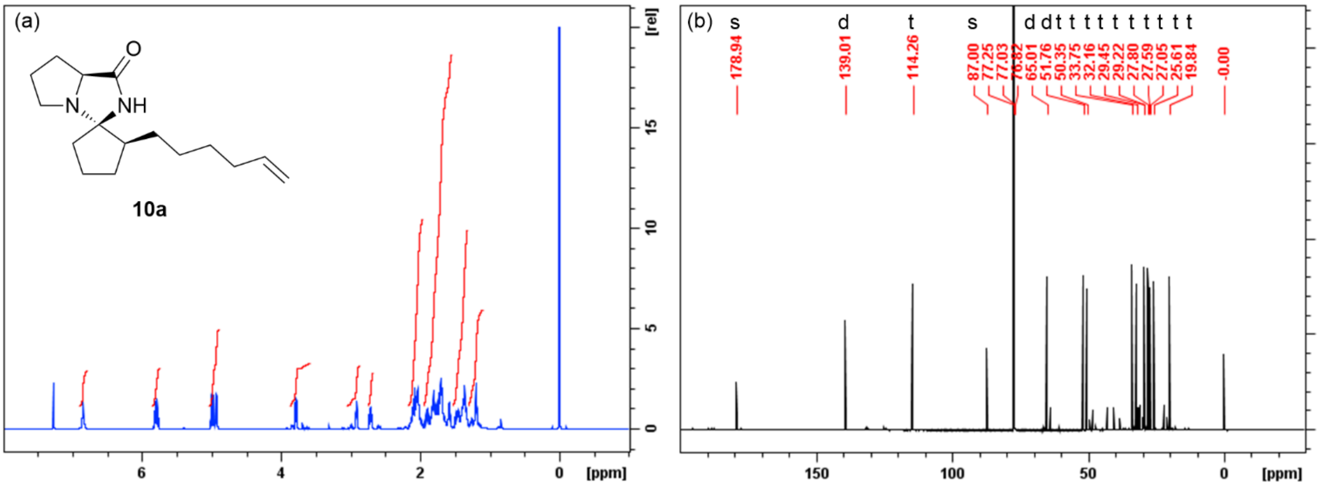

Figure S17. (a) ^{1}H NMR spectrum (600 MHz, $CDCl_3$) and (b) ^{13}C NMR spectrum (151.0 MHz, $CDCl_3$) of (1*S*,2*S*,7*a'S*)-**10a**. The sample contains ca. 10% of the (1*R*,2*R*,7a'*S*)-isomer.

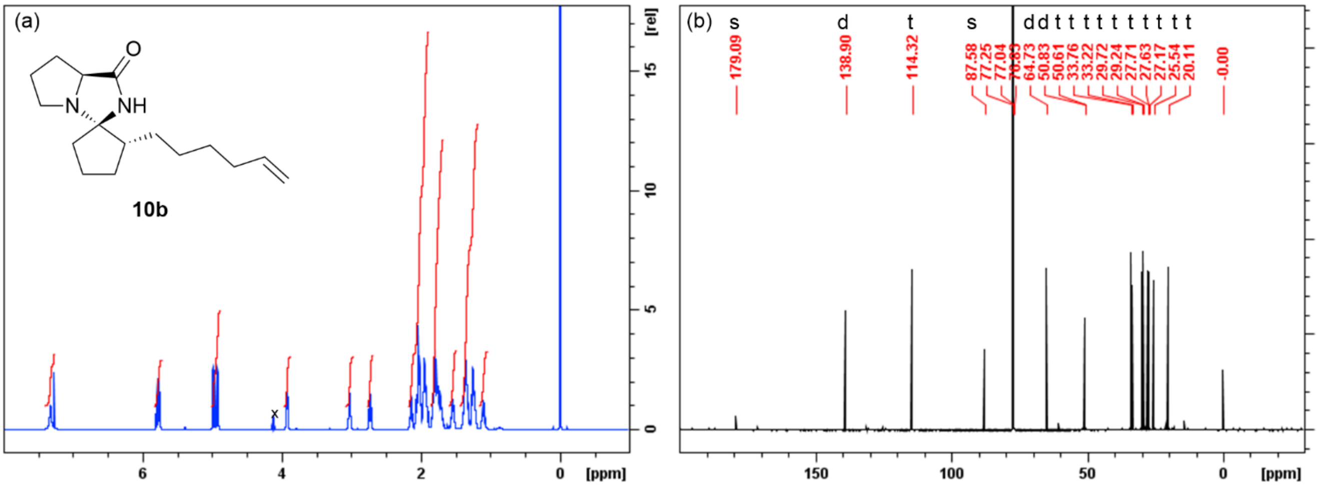

Figure S18. (a) ^{1}H NMR spectrum (600 MHz, $CDCl_3$) and (b) ^{13}C NMR spectrum (151.0 MHz, $CDCl_3$) of (1*S*,2*R*,7*a'S*)-**10b**. The compound contains small amounts of ethyl acetate (labelled as x).

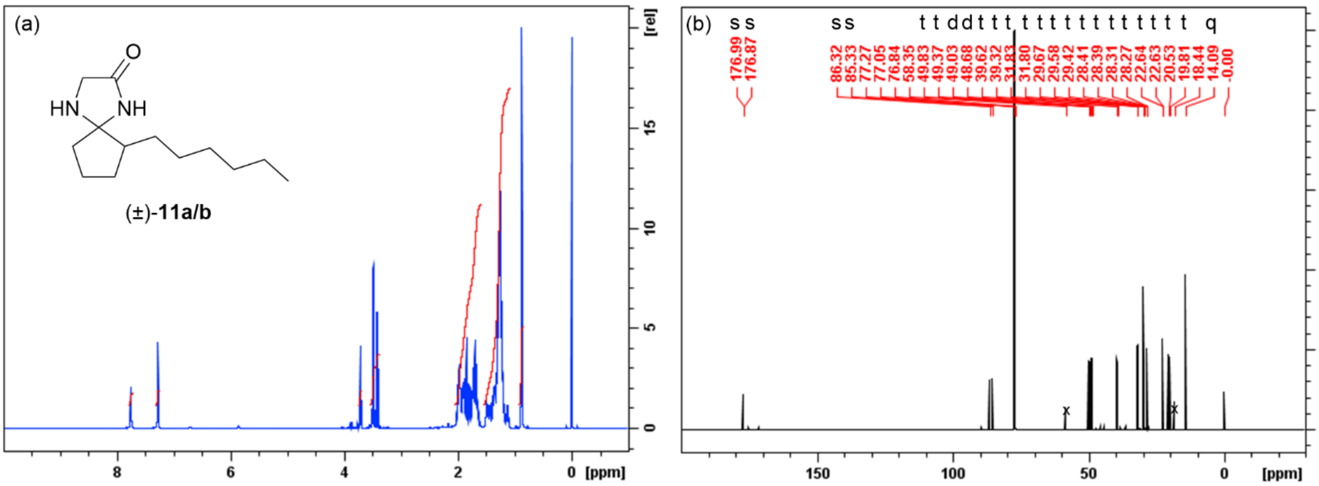

Figure S19. (a) ^{1}H NMR spectrum (600 MHz, $CDCl_3$) and (b) ^{13}C NMR spectrum (150.9 MHz, $CDCl_3$) of (5*RS*,6*RS*)-**11a** and (5*RS*,6*SR*)-**11b**. The compound contains some ethyl acetate (labelled as x)

Tables

Table S1. Integrated ^{1}H NMR signals for the isomerisation of (5*RS*,6*RS*)-**4a** in methanol-d_4 (numerical data for Scheme 6).

Time [min]	(5*RS*,6*RS*)-**4a** (signal at 3.44–3.28 ppm)	(5*RS*,6*SR*)-**4b** (signal at 3.42–3.33 ppm)	Imine ((±)-**12**) (signal at 3.86–3.84 ppm)	Glycinamide (signal at 3.83–3.81 ppm)
0	100.0	0.0	0.0	0.0
32	81.0	18.5	0.5	0.1
106	79.2	20.1	0.7	0.1
167	77.1	22.1	0.7	0.1
322	73.4	25.6	0.9	0.1
1433	56.1	41.9	1.8	0.1
1765	53.0	44.7	2.0	0.2
2055	51.4	46.1	2.2	0.3
5840	44.1	52.1	3.3	0.6
6252	44.4	51.8	3.2	0.6
7360	43.4	52.5	3.3	0.8
8550	43.5	52.1	3.6	0.8
9977	44.1	51.3	3.8	0.9
11581	43.5	51.7	3.8	1.0
15801	43.7	51.3	3.9	1.1
17280	43.8	51.0	4.0	1.2
18734	42.9	51.8	4.2	1.2
20147	43.4	51.3	4.1	1.2
28839	42.4	51.8	4.4	1.4
35932	43.0	51.3	4.1	1.6
46011	43.8	50.2	4.3	1.7
50798	43.3	50.4	4.4	1.9

Table S2. Integrated ^{1}H NMR signals for the isomerisation of (5*RS*,6*SR*)-**4b** in methanol-d_4 (numerical data for Scheme 6).

Time [min]	(5*RS*,6*SR*)-**4b** (signal at 3.42–3.33 ppm)	(5*RS*,6*RS*)-**4a** (signal at 3.44–3.28 ppm)	Imine ((±)-**12**) (signal at 3.86–3.84 ppm)	Glycinamide (signal at 3.83–3.81 ppm)
0	100.0	0.0	0.0	0.0
30	77.1	21.4	0.2	1.3
137	76.1	22.4	0.3	1.3
197	75.1	23.4	0.3	1.2
291	73.5	24.9	0.4	1.2
1402	61.0	36.7	1.0	1.3
1797	58.7	38.8	1.2	1.3
1904	58.5	39.1	1.1	1.3
5809	52.9	43.5	2.1	1.5
7022	52.3	43.8	2.2	1.6
7329	52.4	43.8	2.2	1.5
8580	51.2	44.8	2.4	1.6
10007	52.3	43.9	2.4	1.4
11522	51.4	43.9	2.7	1.9
15769	51.7	43.3	2.9	2.0
17310	51.1	43.9	3.0	2.0
18703	51.3	43.5	3.0	2.1
20177	51.2	43.6	3.1	2.0
28809	51.2	43.2	3.3	2.4
35902	50.5	43.1	3.7	2.6
46042	49.1	44.2	3.7	3.0
50768	48.5	45.0	3.7	2.8

Table S3. Integrated ^{1}H NMR signals for the isomerisation of (1*S*,2*S*,7a'*S*)-**7a** in methanol-d4 (numerical data for Scheme 7).

Time [min]	(1*S*,2*S*,7a'*S*)-**7a** (signal at 3.71–3.79 ppm)	(1*S*,2*R*,7a'*S*)-**7b** (signal at 3.85–3.90 ppm)	(1*R*,2*R*,7a'*S*)-**7c** (signal at 3.80–3.85 ppm)	*L*-prolinamide (signal at 3.65–3.71 ppm)
0	100.0	0.0	0.0	
32	75.6	4.1	11.8	8.5
283	58.5	9.5	23.2	8.8
445	55.9	13.0	22.0	9.1
1337	47.3	24.1	18.8	9.8
1618	45.8	26.4	18.1	9.7
2609	41.2	31.8	16.6	10.4
2768	40.7	32.5	16.3	10.5
3578	38.2	35.4	15.6	10.8
4687	35.9	38.2	14.4	11.4
5633	34.2	39.7	14.0	12.2
7518	31.4	42.0	13.0	13.6
9990	28.5	43.3	12.2	15.9
10422	28.1	44.3	11.7	15.9
11666	27.0	45.1	11.1	16.8
13766	25.1	45.8	10.5	18.6
15904	23.2	47.1	9.4	20.3
30060	17.5	43.4	7.4	31.6
34408	16.8	41.5	7.4	34.3
40162	15.7	39.6	6.9	37.8
42013	15.6	38.4	7.0	39.0
44982	15.2	37.6	6.8	40.5
46438	15.0	37.3	6.6	41.1
51700	14.1	36.1	6.0	43.8
60337	13.2	33.9	5.5	47.4
64627	12.3	33.5	4.7	49.6
71859	12.6	31.8	5.1	50.4
82177	11.8	30.0	4.9	53.2

Table S4. Integrated ^{1}H NMR signals for the isomerisation of (1*S*,2*R*,7a'*S*)-**7b** in methanol-d4 (numerical data for Scheme 7).

Time [min]	(1*S*,2*S*,7a'*S*)-**7a** (signal at 3.71–3.79 ppm)	(1*S*,2*R*,7a'*S*)-**7b** (signal at 3.85–3.90 ppm)	(1*R*,2*R*,7a'*S*)-**7c** (signal at 3.80–3.85 ppm)	*L*-prolinamide (signal at 3.65–3.71 ppm)
0	0	100	0	0
35	3.2	92.7	3.8	0
583	5.6	88.3	5.3	0
1467	8.7	84.8	6.1	0
2038	10.5	82.1	6.9	0
7300	20.2	66.3	9.9	3.6
19418	21.9	53.9	10.6	13.7
20226	21.9	53.1	10.6	14.5
21760	21.5	52.4	10.3	15.7
25992	20.3	52.1	9.3	18.3
27458	20.3	49.6	9.9	20.3
37777	18.4	45.6	9.1	26.8
46958	17.0	43.3	8.2	31.5
67541	14.6	38.7	7.4	39.3
79541	13.5	35.1	7.6	43.8

Table S5. Integrated ^{1}H NMR signals for the isomerisation of (1*R*,2*R*,7a'*S*)-**7c** in methanol-d_4 (numerical data for Scheme 7).

Time [min]	(1*S*,2*S*,7a'*S*)-**7a** (signal at 3.71–3.79 ppm)	(1*S*,2*R*,7a'*S*)-**7b** (signal at 3.85–3.90 ppm)	(1*R*,2*R*,7a'*S*)-**7c** (signal at 3.80–3.85 ppm)	*L*-prolinamide (signal at 3.65–3.71 ppm)
0	0	0	100	0
32	33.2	2.9	63.0	0.0 [1]
107	61.9	5.2	31.1	0.1 [1]
217	63.9	8.4	26.3	0.3 [1]
249	63.4	8.6	25.7	0.3 [1]
501	59.3	14.0	24.0	0.7 [1]
1287	52.4	23.2	22.1	1.7 [1]
1959	49.7	28.4	19.9	2.5 [1]
2751	47.6	32.2	18.1	3.3 [1]
3648	45.3	35.5	17.1	4.2 [1]
7041	35.7	42.8	14.6	6.8
8892	33.1	46.1	13.3	7.5
9945	31.7	47.6	12.7	8.0
14529	27.1	50.0	11.3	11.7
23021	21.9	53.5	8.2	16.4
30155	20.4	49.7	8.5	21.4
38857	19.8	45.9	8.7	25.6
48640	18.2	45.2	7.8	28.8
57764	17.5	43.5	7.3	31.7
78272	15.7	40.3	6.4	37.6

[1] extrapolated (not measured) data.

Table S6. Average dynamic headspace concentrations of (±)-**1** with standard deviations (in parentheses) measured on cotton after line drying for 1 day (numerical data for Figure 6).

Time [min]	(±)-**1** (Reference) [ng L^{-1}]	(±)-**1** from **4a/b** [ng L^{-1}]	(±)-**1** from (5*RS*,6*RS*)-**4a** [ng L^{-1}]	(±)-**1** from (5*RS*,6*SR*)-**4b** [ng L^{-1}]	(±)-**1** from **5a–d** [ng L^{-1}]	(±)-**1** from (3*S*,5*R*,6*R* and 3*S*,5*S*,6*S*)-**6a/c** [ng L^{-1}]
30	0.2 (±0.1)	1.5 (±0.4)	3.1 (±0.2)	3.1 (±0.3)	1.0 (±0.5)	1.4 (±0.3)
90	0.6 (±0.3)	5.3 (±1.4)	5.1 (±0.4)	4.6 (±0.3)	5.6 (±0.9)	2.7 (±0.1)
150	0.6 (±0.3)	7.6 (±2.8)	6.3 (±0.4)	5.6 (±0.8)	7.9 (±4.7)	3.4 (±0.2)
210	0.5 (±0.3)	7.4 (±2.6)	7.3 (±0.2)	6.0 (±1.1)	6.2 (±2.6)	3.6 (±0.2)
270	0.7 (±0.4)	7.2 (±1.5)	7.8 (±0.3)	6.2 (±0.7)	5.0 (±1.4)	3.5 (±0.3)
330	0.4 (±0.3)	6.7 (±1.5)	8.1 (±0.1)	6.2 (±0.7)	4.3 (±0.2)	3.5 (±0.4)
390	0.4 (±0.3)	6.1 (±1.2)	8.8 (±0.2)	6.1 (±0.7)	3.4 (±0.2)	3.2 (±0.3)
450	0.4 (±0.3)	5.9 (±1.2)	9.1 (±0.1)	6.1 (±0.7)	2.9 (±0.3)	3.3 (±0.0)
Sum	3.7 (ref.)	47.7 (×13)*	55.5 (×15)*	44.0 (×12)*	36.2 (×10)*	24.6 (×7)*

Time [min]	(±)-**1** from (3*S*,5*S*,6*R* or 3*S*,5*R*,6*S*)-**6b/d** [ng L^{-1}]	(±)-**1** from **7a–d** [ng L^{-1}]	(±)-**1** from (1*S*,2*S*,7a'*S*)-**7a** [ng L^{-1}]	(±)-**1** from (1*S*,2*R*,7a'*S*)-**7b** [ng L^{-1}]	(±)-**1** from (1*R*,2*R*,7a'*S*)-**7c** [ng L^{-1}]	(±)-**1** from **8a–c** [ng L^{-1}]
30	0.6 (±0.1)	11.8 (±5.5)	16.1 (±12.9)	9.0 (±2.5)	14.7 (±3.4)	1.1 (±0.1)
90	1.5 (±0.2)	31.9 (±25.6)	59.4 (±61.3)	30.9 (±14.6)	61.8 (±43.5)	2.3 (±0.1)
150	1.9 (±0.1)	39.9 (±34.7)	63.3 (±55.5)	42.4 (±20.5)	72.6 (±49.4)	2.7 (±0.6)
210	1.9 (±0.1)	40.1 (±31.3)	59.2 (±43.6)	43.6 (±18.3)	65.3 (±39.5)	3.0 (±1.0)
270	1.9 (±0.1)	35.9 (±24.5)	53.6 (±38.0)	39.4 (±15.6)	57.8 (±30.2)	2.5 (±1.0)
330	1.8 (±0.1)	31.9 (±19.5)	48.4 (±32.3)	37.1 (±12.2)	37.9 (±32.9)	2.3 (±0.7)
390	1.8 (±0.2)	30.0 (±15.0)	43.0 (±27.9)	33.8 (±9.4)	43.0 (±17.6)	2.0 (±0.4)
450	1.7 (±0.2)	21.9 (±12.1)	40.0 (±24.0)	33.2 (±8.1)	40.1 (±15.5)	1.7 (±0.4)
Sum	13.2 (×4)*	243.4 (×66)*	383.1 (×104)*	269.4 (×73)*	393.2 (×106)*	17.5 (×5)*

* factor of increase with respect to the reference.

Table S7. Average dynamic headspace concentrations of (±)-**1** with standard deviations (in parentheses) measured on cotton after line drying for 3 days (numerical data for Figure 6).

Time [min]	(±)-**1** (Reference) [ng L^{-1}]	(±)-**1** from **4a/b** [ng L^{-1}]	(±)-**1** from (5*RS*,6*RS*)-**4a** [ng L^{-1}]	(±)-**1** from (5*RS*,6*SR*)-**4b** [ng L^{-1}]	(±)-**1** from **5a–d** [ng L^{-1}]	(±)-**1** from (3*S*,5*R*,6*R* and 3*S*,5*S*,6*S*)-**6a/c** [ng L^{-1}]
30	0.4 (±0.1)	1.9 (±1.5)	3.1 (±0.1)	2.8 (±0.3)	0.7 (±0.2)	1.3 (±0.0)
90	0.9 (±0.3)	6.7 (±1.3)	7.0 (±0.2)	7.8 (±0.2)	4.8 (±0.2)	4.1 (±0.2)
150	0.9 (±0.2)	10.1 (±3.9)	9.4 (±0.3)	11.3 (±2.9)	5.3 (±1.8)	6.8 (±0.4)
210	0.8 (±0.5)	11.6 (±5.4)	11.8 (±0.6)	13.3 (±4.1)	4.6 (±1.0)	8.4 (±1.5)
270	0.7 (±0.5)	9.1 (±6.0)	12.6 (±0.0)	12.0 (±2.9)	4.0 (±0.4)	7.8 (±2.4)
330	0.7 (±0.5)	8.0 (±4.9)	14.1 (±0.2)	11.1 (±1.9)	3.1 (±0.3)	7.1 (±2.4)
390	0.7 (±0.6)	7.2 (±4.2)	13.5 (±0.8)	9.9 (±0.9)	2.5 (±0.0)	6.7 (±2.5)
450	0.6 (±0.4)	6.0 (±3.3)	14.0 (±0.6)	10.2 (±0.2)	2.2 (±0.0)	5.7 (±2.1)
Sum	5.7 (ref.)	60.8 (×11)*	85.6 (×15)*	78.4 (×14)*	27.3 (×5)*	47.8 (×8)*

Time [min]	(±)-**1** from (3*S*,5*S*,6*R* or 3*S*,5*R*,6*S*)-**6b/d** [ng L^{-1}]	(±)-**1** from **7a–d** [ng L^{-1}]	(±)-**1** from (1*S*,2*S*,7a'*S*)-**7a** [ng L^{-1}]	(±)-**1** from (1*S*,2*R*,7a'*S*)-**7b** [ng L^{-1}]	(±)-**1** from (1*R*,2*R*,7a'*S*)-**7c** [ng L^{-1}]	(±)-**1** from **8a–c** [ng L^{-1}]
30	0.7 (±0.1)	6.2 (±1.4)	5.1 (±1.7)	11.1 (±5.6)	5.8 (±1.1)	0.3 (±0.1)
90	2.1 (±0.3)	15.5 (±8.2)	24.8 (±27.6)	32.0 (±6.7)	21.0 (±8.6)	1.3 (±0.8)
150	2.7 (±0.1)	25.0 (±16.3)	34.9 (±37.0)	50.2 (±16.9)	31.2 (±16.0)	1.7 (±1.5)
210	3.2 (±0.2)	27.8 (±19.7)	30.8 (±26.7)	55.2 (±21.9)	31.8 (±17.6)	1.6 (±1.2)
270	2.7 (±0.1)	27.6 (±19.9)	26.4 (±20.9)	52.9 (±21.8)	28.5 (±16.4)	1.3 (±0.9)
330	2.6 (±0.1)	26.3 (±15.8)	22.4 (±16.6)	46.8 (±16.3)	25.0 (±14.0)	1.2 (±0.9)
390	2.5 (±0.1)	23.0 (±15.2)	19.6 (±14.1)	42.6 (±13.9)	17.3 (±16.2)	1.0 (±0.7)
450	2.4 (±0.0)	21.3 (±12.7)	16.8 (±11.4)	38.2 (±9.8)	19.5 (±10.5)	0.9 (±0.6)
Sum	18.8 (×3)*	172.8 (×30)*	180.8 (×32)*	329.1 (×58)*	180.1 (×32)*	9.2 (×2)*

* factor of increase with respect to the reference.

Table S8. Average dynamic headspace concentrations of (±)-**2** and (±)-**3** with standard deviations (in parentheses) measured on cotton after line drying for 1 day (numerical data for Figure 7).

Time [min]	(±)-**2** (Reference) [ng L^{-1}]	(±)-**2** from (5*RS*,6*RS*)-**9a** [ng L^{-1}]	(±)-**2** from (5*RS*,6*SR*)-**9b** [ng L^{-1}]	(±)-**2** from (1*S*,2*S*,7a'*S*)-**10a** [ng L^{-1}]	(±)-**2** from (1*S*,2*R*,7a'*S*)-**10b** [ng L^{-1}]	(±)-**3** (Reference) [ng L^{-1}]	(±)-**3** from **11a/b** [ng L^{-1}]
30	0.2 (±0.1)	1.0 (±0.1)	1.1 (±0.1)	5.5 (±0.5)	2.2 (±1.5)	0.7 (±0.1)	1.1 (±0.3)
90	0.4 (±0.1)	3.6 (±0.4)	4.2 (±0.0)	28.1 (±1.2)	19.2 (±5.8)	1.4 (±0.1)	5.9 (±1.6)
150	0.9 (±0.1)	6.0 (±1.8)	7.1 (±0.5)	62.9 (±4.3)	36.6 (±11.3)	2.0 (±0.4)	9.8 (±1.0)
210	0.8 (±0.0)	7.2 (±1.5)	7.4 (±1.1)	83.5 (±5.9)	42.6 (±10.7)	1.6 (±0.3)	12.5 (±0.9)
270	1.0 (±0.6)	7.5 (±1.1)	7.5 (±1.6)	86.7 (±4.6)	44.0 (±9.1)	1.1 (±0.1)	14.0 (±1.7)
330	0.6 (±0.1)	7.4 (±0.8)	7.0 (±1.5)	83.2 (±2.5)	45.5 (±8.9)	0.9 (±0.1)	13.3 (±0.7)
390	2.5 (±3.1)	7.1 (±0.8)	6.9 (±1.5)	74.3 (±1.9)	43.7 (±7.3)	0.7 (±0.1)	12.6 (±0.1)
450	0.4 (±0.2)	6.7 (±0.5)	6.6 (±1.0)	66.8 (±2.5)	42.5 (±8.0)	0.6 (±0.1)	11.9 (±0.1)
Sum	6.8 (ref.)	46.5 (×7)*	47.7 (×7)*	491.1 (×72)*	276.4 (×41)*	8.9 (ref.)	81.1 (×9)*

* factor of increase with respect to the reference.

Table S9. Average dynamic headspace concentrations of (±)-**2** and (±)-**3** with standard deviations (in parentheses) measured on cotton after line drying for 3 days (numerical data for Figure 7).

Time [min]	(±)-**2** (Reference) [ng L^{-1}]	(±)-**2** from (5*RS*,6*RS*)-**9a** [ng L^{-1}]	(±)-**2** from (5*RS*,6*SR*)-**9b** [ng L^{-1}]	(±)-**2** from (1*S*,2*S*,7a'*S*)-**10a** [ng L^{-1}]	(±)-**2** from (1*S*,2*R*,7a'*S*)-**10b** [ng L^{-1}]	(±)-**3** (Reference) [ng L^{-1}]	(±)-**3** from **11a/b** [ng L^{-1}]
30	0.1 (±0.2)	0.7 (±0.1)	0.5 (±0.1)	0.8 (±0.3)	0.7 (±0.1)	0.3 (±0.0)	2.0 (±1.4)
90	0.1 (±0.1)	3.5 (±0.1)	1.7 (±2.2)	3.9 (±4.6)	10.9 (±4.0)	0.5 (±0.1)	8.3 (±0.8)
150	0.5 (±0.2)	8.0 (±0.2)	7.8 (±1.5)	8.2 (±10.2)	26.4 (±5.0)	1.0 (±0.1)	28.1 (±18.0)
210	0.8 (±0.2)	11.4 (±0.1)	10.4 (±0.8)	12.9 (±16.1)	42.1 (±15.4)	1.3 (±0.3)	26.4 (±10.1)
270	0.8 (±0.3)	12.4 (±0.5)	11.3 (±2.1)	15.7 (±19.7)	48.9 (±18.0)	1.3 (±0.1)	26.6 (±4.9)
330	1.5 (±1.1)	12.2 (±0.0)	11.0 (±3.3)	18.9 (±23.9)	47.1 (±17.1)	0.9 (±0.0)	42.4 (±1.6)
390	0.6 (±0.2)	11.5 (±0.9)	9.8 (±2.3)	20.9 (±26.0)	45.3 (±16.0)	1.0 (±0.2)	22.1 (±1.8)
450	0.6 (±0.1)	10.7 (±1.7)	9.5 (±1.4)	22.1 (±27.3)	42.2 (±14.3)	0.8 (±0.1)	17.0 (±0.4)
Sum	5.1 (ref.)	70.3 (×14)*	62.0 (×12)*	103.3 (×20)*	263.5 (×52)*	7.1 (ref.)	172.9 (×24)*

* factor of increase with respect to the reference.
